# Novel CSF Biomarkers Tracking Autoimmune Inflammatory and Neurodegenerative Aspects of CNS Diseases

**DOI:** 10.3390/diagnostics13010073

**Published:** 2022-12-27

**Authors:** Elisabeth Kapaki, Aigli G. Vakrakou, Fotini Boufidou

**Affiliations:** 1 Neurochemistry and Biological Markers Unit, 1st Department of Neurology, School of Medicine, National and Kapodistrian University of Athens, Eginition Hospital, 74, Vass. Sophias Ave., 11528 Athens, Greece; 2 Institute of Neuropathology, University Medical Center Göttingen, 37075 Göttingen, Germany

**Keywords:** cerebrospinal fluid, biomarkers, neuroinflammation, neurodegeneration

## Abstract

The accurate diagnosis of neuroinflammatory (NIDs) and neurodegenerative (NDDs) diseases and the stratification of patients into disease subgroups with distinct disease-related characteristics that reflect the underlying pathology represents an unmet clinical need that is of particular interest in the era of emerging disease-modifying therapies (DMT). Proper patient selection for clinical trials and identifying those in the prodromal stages of the diseases or those at high risk will pave the way for precision medicine approaches and halt neuroinflammation and/or neurodegeneration in early stages where this is possible. Towards this direction, novel cerebrospinal fluid (CSF) biomarker candidates were developed to reflect the diseased organ’s pathology better. Μisfolded protein accumulation, microglial activation, synaptic dysfunction, and finally, neuronal death are some of the pathophysiological aspects captured by these biomarkers to support proper diagnosis and screening. We also describe advances in the field of molecular biomarkers, including miRNAs and extracellular nucleic acids known as cell-free DNA and mitochondrial DNA molecules. Here we review the most important of these novel CSF biomarkers of NIDs and NDDs, focusing on their involvement in disease development and emphasizing their ability to define homogeneous disease phenotypes and track potential treatment outcomes that can be mirrored in the CSF compartment.

## 1. Introduction

One hundred and thirty-one years since Heinrich Quincke carried out the first lumbar puncture and attempted cerebrospinal fluid (CSF) cytological examination, significant progress has been made in CSF analysis and diagnostics. Over the years, this “third circulation” or “the vital spirit” has become a readily available and important means of diagnosing and studying diseases affecting the nervous system with such accuracy that is considered—not unfairly—as the “liquid biopsy” of the brain. Nowadays, successful scientific and technical efforts have resulted in overcoming laboratory “barriers” in CSF analysis, such as the low concentrations of diagnostically important molecules and the mixture of blood vs. brain-derived proteins in the intrathecal compartment, rendering CSF analysis a powerful diagnostic tool at any time an immune-inflammatory or a neurodegenerative disease is suspected [1].

With regard to autoimmune-demyelinating disorders, with multiple sclerosis (MS) the major representative, CSF’s contribution to diagnosis has long been appreciated. The recognition of “oligoclonal bands” (OBs)—known since the 1970s—along with the determination of intrathecal immunoglobulin synthesis is part of daily clinical practice [2]. The fluid biomarker field in MS is developing rapidly reflecting unmet needs in three major fields, that of diagnosis, progression of disability, and treatment outcomes. In this respect, kappa (K) and lambda (L) free light chains (FLC) are emerging biomarkers for intrathecal immunoglobulin synthesis, while neurofilaments (NF) are being investigated as biomarkers of disease progression and response to therapy. However, additional biomarkers are needed to cover broader pathological processes (such as oligodendrocytes apoptosis, neuronal death, remyelination, microglial activation, and neuroaxonal damage) to stratify MS patients into subgroups that reflect disease activity and disability accumulation, with the aim of opening up new possibilities for timely targeted therapies. Autoantibodies (Abs), also used as biomarkers for the diagnosis of recently recognized autoimmune disorders, have been added to an ever-growing list of Abs responsible for autoimmune/paraneoplastic encephalopathies, further advancing CSF diagnostics [3].

In the last 30 years, great advances have also been made in the field of biological markers of neurodegeneration, particularly in dementia-related disorders, mainly Alzheimer’s disease (AD). Biomarker discovery has been based on the identification of proteins proven to be closely associated with relevant biological processes, and cell or tissue pathologies. The pathophysiology of AD, the most common form of dementia, involves the extracellular aggregation of misfolded Aβ species, and the accumulation of tau proteins into the neurons, leading to synaptic loss, axonal damage, neurodegeneration, and finally cell death. Established CSF biomarkers already in use indicative of Aβ pathology are CSF Aβ42 and/or Aβ42/Aβ40 ratio, while tangle-related pathology can be captured by measurement of CSF phospho-tau protein (p-tau) levels. Biomarkers indicative of neurodegeneration in general, include CSF total tau (t-tau), neurofilaments, and 14-3-3 protein, among others [4,5]. All these biomarkers have been evaluated in the (A/T/N) classification system proposed by the National Inst. on Aging and Alzheimer’s Association Research Framework [6]. However, additional biomarkers with molecular specificity are needed (such as alpha-synuclein, TPD-43, and progranulin) to accurately diagnose other neurodegenerative and/or vascular dementias, along with other nonspecific biomarkers of neurodegeneration. Moreover, microRNA analysis and molecular biomarkers are continuously explored, expanding the potential of CSF diagnostics.

The objective of the present study is not aspiring to be an exhaustive review of the ever-growing literature but to highlight the most important substances having the potential to emerge as novel CSF biomarkers tracking neurodegenerative and/or autoimmune-inflammatory aspects of CNS diseases.

## 2. Biomarkers Tracking Inflammatory Aspects

### 2.1. Intrathecal Free Light Chain Synthesis

The most reliable biomarker for MS diagnosis is the presence of OBs that correspond to immunoglobulins produced by plasma cells and are primarily identified in CSF, compared to serum, indicative of their intrathecal synthesis. Antigens involved in the stimulation, activation, and differentiation of B cells in antibody-secreting plasma cells in MS are largely unknown [7]. The high clinical utility of OBs in MS is reflected by the incorporation of this biomarker in the 2017 McDonald criteria [2]. However, they are not only used in daily clinical practice as a diagnostic but also as a prognostic biomarker for future progression to definite MS, especially in patients with clinically isolated syndrome (CIS) and radiologically isolated syndrome (RIS), with a hazard ratio that reaches the value of 10 [8,9]. Nevertheless, it lacks high specificity.

More in-depth knowledge of immunoglobulin biology reveals that light chains are produced as either kappa or lambda isotypes during immunoglobulin synthesis, in approximately 20% excess over the heavy chains [10]. Finally, this plasma-cell “byproduct” is secreted as kappa free light chains (KFLC) and lambda (LFLC) isotypes, respectively. Excessive intrathecal IgG production, the most prominent immunological hallmark in MS, eventually results in increased CSF FLC levels in MS, which is already known since the 1980s [11,12,13]. However, it was the development of automated nephelometric and turbitometric assays which made possible a reliable quantification of FLC and advocated their diagnostic utility [14]. Particularly for KFLC analysis, encouraging results have shown high sensitivity and specificity for CIS progression in clinically definite MS [13,15,16], making this fully automated test to glare as a promising novel biomarker for quantitative intrathecal IgG synthesis that could even replace technically demanding and rater-dependent OBs in MS [17]. Initial studies have shown that the KFLC index greater than 5.9 had a 96% diagnostic sensitivity for MS [13]. Saadeh et al. [17] found that quantitative measurement of CSF KCSF using a cutoff of 0.10 mg/dL is not significantly different from the performance of positive OB testing (no serum coupling in this study). Indeed, KCSF (kappa free light chain of cerebrospinal fluid) vs. OBs sensitivities were 78.6% for both (*p* > 0.99) and specificities 87.1% vs. 89.4%, respectively [18].

Meanwhile, multiple attempts have been made for the optimal use of CSF and blood KFLC levels alone or appropriately correlated with markers of blood–brain barrier (BBB) functionality and a consensus regarding a reference range and cut-off values able to distinguish between patients and healthy population [14,19,20,21,22,23,24]. A more sophisticated, non-linear approach that incorporates molecule diffusion and CSF flow rates for the identification of KFLC intrathecal synthesis is needed. Towards this attempt, a theoretically and empirically proposed hyperbolic function formula in correspondence to former schemes for IgG, IgM, and IgA immunoglobulins was introduced in 2019 by Reiber et al. [25]. Although having shown promising results in increasing specificity for MS diagnosis, further evaluation in larger cohorts and multicenter studies is needed.

### 2.2. YKL-40

YKL-40, a glycoprotein, also known as chitinase 3-like protein 1, possesses an important role in the remodeling of the extracellular matrix and is closely related to the inflammatory response observed in various cell types, such as synovial fibroblasts, chondrocytes, macrophages, and neutrophils [26,27]. In the CNS, YKL-40 is mostly expressed by astrocytes but also by activated macrophages and microglia, especially under inflammatory conditions [27,28]. A cross-talk between macrophages and astrocytes has been identified, whereby macrophage-released cytokines cause astrocytes to produce YKL-40, which alters their morphology and impairs their motility [28].

YKL-40 seems to be up-regulated in inflamed tissues and exhibits an altered expression in neuroinflammatory diseases, mainly in MS. In brain specimens from MS patients, YKL-40 is expressed by numerous reactive astrocytes located in white matter plaques, and the normal-appearing white matter (NAWM) [29]. Apart from astrocytes, YKL-40 was also strongly expressed by CD68 + cells, a general macrophage/microglia marker, in white matter plaques, NAWM, as well as perivascular spaces [30]. Importantly, YKL-40, found by proteomic analysis of CSF samples, has been suggested as a useful biomarker for predicting disease progression from CIS to classical relapsing-remitting MS (RRMS) [31]. CSF YKL-40 levels are predominantly increased in progressive compared to relapsing MS and are associated with a higher risk of disability accumulation and correlated with MRI measures indicative of spinal cord atrophy [32].

Regarding AD, it has been shown that the evolution of neuritic plaques is closely associated with activated phagocytic microglia [33]. CSF YKL-40 levels are closely correlated with CSF t-tau and p-tau levels, indicative of the role of YKL-40 in tracking the neuroinflammation secondary to neurodegeneration [34]. A promising study showed that YKL-40 CSF levels differed between prodromal and established AD [35]. Importantly, this biomarker could be helpful in the prediction of the conversion of mild cognitive impairment (MCI) to AD, especially in the presence of the *APOE* ε4 allele [36]. Another study confirmed these results showing that the YKL-40 levels were increased in the CSF of MCI-AD patients compared to those with stable MCI; therefore, authors suggested that this protein could represent a potential prognostic biomarker for the progression of MCI to clinical AD [37]. Nevertheless, CSF YKL-40 could not discriminate well among AD from non-AD dementias, e.g., dementia with Lewy bodies (DLB) and frontotemporal dementia (FTD). Among other neurodegenerative diseases, higher levels of CSF YKL-40 have been shown in amyotrophic lateral sclerosis (ALS) and sporadic Creutzfeldt–Jakob disease (CJD) patients compared to healthy individuals [38,39]. Further studies are needed to better elucidate the role of YKL-40 as a CSF surrogate biomarker of neurodegeneration-related innate immunity activation in the pre-symptomatic/initial stages of AD continuum as well as in other neurodegenerative diseases, possibly in combination with established AD biomarkers.

### 2.3. sTREM2

Triggering receptor expressed on myeloid cells 2 (TREM2) is an innate immunity receptor expressed by microglial cells. Microglial activation leads to cleavage and production of its fragment soluble TREM2 (sTREM2), which is then able to be measured in the CSF, serving as a surrogate marker of microglial activity. In MS pathophysiology, Piccio et al. [40] first described increased sTREM2 in CSF (assessed by enzyme-linked immunosorbent assay—ELISA) in active MS patients. In this study, CSF monocytes were found to express TREM2, and it was highly expressed on myelin-laden macrophages in actively demyelinated lesions from four autopsied MS patients. Towards this, the CSF levels of sTREM2 showed significant correlations with inflammatory cytokines IL-8, granulocyte colony-stimulating factor, and IL-5, supporting the role of microglial/macrophage activation in the inflammatory reactions during active disease stages [41]. sTREM2 levels are also amenable to drug-related disease manipulation as treatment with natalizumab or mitoxantrone normalizes CSF sTREM2 concentrations [42].

TREM2 gene mutations are of particular interest because they are associated with an increase in the risk for AD, and especially those carrying the R47H genetic variant have increased risk with Odds Ratios similar to those of APOE [43,44]. TREM2 risk variant carriers have been shown to have higher levels of sTREM2 than controls and non-carriers. Regarding early-stage AD, sTREM2 was associated with tau-related neurodegeneration more consistently than with amyloid-β pathology [45,46]. The levels of CSF sTREM2 were significantly higher in AD patients compared to cognitively normal individuals and displayed significant relationships with p-tau-181 and t-tau [40,47,48]. Nevertheless, other studies showed no significant changes in levels of CSF sTREM2 between controls and AD patients [49,50]. Suárez-Calvet et al. [45] measured sTREM2 levels across the AD spectrum and found that CSF sTREM2 was lowest in controls and preclinical AD, peaked in MCI-AD, and then declined slightly in AD dementia. CSF sTREM2 levels correlated better with CSF t-tau and p-tau levels than Aβ42 levels, suggesting that elevated sTREM2 levels occur later in the course of the AD process [51]. The most intriguing aspect is that sTREM2 captures microglial activation that closely relates to a disease stage-dependent fashion in AD, with the highest levels in early symptomatic stages depicting signs of microglia activation secondary to neuronal degeneration [45,46].

A recent study suggests a model in which Aβ and microglial activation as two partially independent processes that, when acting synergistically, lead to neocortical tau pathology. Pascoal et al. (2021), by using novel positron emission tomography (PET) brain imaging for capturing microglial, amyloid, and tau pathology, found that microglia activation correlates with tau pathology progression according to the stereotypical pattern of propagation of tau tangles from the transentorhinal/entorhinal to sensorimotor cortices in AD (known as Braak stages) [52]. CSF sTREM, correlated with [^11^C]PBR28 PET imaging (indicative of microglial activation), suggesting that sTREM2 could be a novel marker for in vivo microglia activation [52].

Other studies evaluating CSF sTREM2 have revealed increased levels in Parkinson’s disease (PD) vs. controls that also correlated with CSF α-synuclein [53] as well as in *CHMP2B* and *GRN* mutation carriers of FTD patients [54] and in CJD patients in whom levels correlated with CSF t-tau, 14.3.3 protein and YKL-40 [55].

### 2.4. Soluble CD136 and CXCL13

sCD163, a marker of activated microglia and macrophages [56]; and CXCL13, necessary for the development of B-cell follicles and secondary lymphoid structures, as a well-known B-cell chemoattractant, produced mainly by follicular dendritic cells and possibly by activated macrophages and microglial cells [57,58]; are promising CSF markers that are under investigation for future use in MS diagnostics [59]. Especially, CSF CXCL13 levels were found to be increased in neuromyelitis optica (NMO) compared with MS patients and were related to the NMO disease activity indicated by relapse rate and Expanded Disability Status Scale (EDSS) scores [60].

### 2.5. IL-6

A hot topic in the era of fluid-based biomarkers is the identification of those able to distinguish between NMO and MS patients. One potential candidate biomarker for such differentiation is CSF IL-6 levels. IL-6 is a proinflammatory cytokine, mainly produced by macrophages/monocytes and some activated B cells, and is considered a Th17 polarizing cytokine, thus fostering subsequent autoantibody production [61,62]. NMO relapses are associated with highly increased IL-6 levels vs. MS patients. Importantly, IL-6 correlates with various clinical parameters of NMO, such as the length of myelitis and disease severity scores, particularly in drug-naive patients, while it correlates with markers of glial damage, such as glial fibrillary acidic protein [63,64,65,66].

Other potential CSF biomarkers discriminating MS from NMO are CSF complement components (e.g., C5a and sC5b-9) as well as Th2 and Th17-related cytokines/chemokines (e.g., IL-17, IL-13) [67,68]. Most of them have been analyzed in only a few studies, and further validation is required. Finally, regarding discrimination between myelin oligodendrocyte glycoprotein antibody disease (MOGAD) and NMO, which share a lot of common pathways, CSF biomarker(s) are still lacking.

### 2.6. Monocyte Chemoattractant Protein-1 (MCP-1/CCL2)

Monocyte chemoattractant protein-1 (MCP-1) is an important chemokine for the recruitment of monocytes and macrophages to the CNS through its interaction with its receptor CCR2. Tissue macrophages and microglia cells in active white matter lesions of MS patients have been found to overexpress CCR2. CCL2 expression was defined as activated astrocytes, pointing to their role as active players in orchestrating the inflammatory milieu in MS white matter lesions [69,70]. MCP-1/CCL2 levels in MS have been found to be decreased in the CSF, especially during active disease stages, and correlated with indices of intrathecal IgG production and CSF levels of neurofilament light protein (NFL) [71].

MCP-1 has been also reported significantly increased in prodromal AD compared to healthy controls and correlated with a short time interval to cognitive decline and development of dementia. In combination with CSF Aβ42 and t-tau and p-tau protein levels, it may be a potentially useful biomarker for monitoring disease progression [72].

### 2.7. Glial Fibrillary Acidic Protein (Astrogliosis Marker)

Glial fibrillary acidic protein (GFAP) is a cytoskeletal protein expressed by mature astrocytes and is widely used as a cell type marker in brain histopathology [73]. Increased CSF GFAP levels in MS are predictive of disability reached 8–10 years later [74]. Reactive astrocytes have been found to overexpress GFAP in the plaques of MS patients [75,76]. Patients with secondary progressive MS (SPMS) displayed higher CSF GFAP levels than those with RRMS [77]. Additionally, higher GFAP CSF levels were associated with poor ambulation and greater disabilities in MS [77,78].

GFAP, when measured in CSF, exhibited higher values in patients with NMO spectrum disorders (NMOSD) when compared to patients with MS or healthy controls [79,80,81,82,83,84,85]. S100B protein, another marker of astrocytic damage, showed a similar trend but with less statistical strength [80]. Other studies have demonstrated that the values of GFAP and S100B are lower in seronegative patients when compared to AQP4-IgG-positive NMOSD patients [82]. As a marker that discriminates between NMOSD and MS, the increase in GFAP values was proposed to be a supportive criterion for NMOSD diagnosis [86]. However, the utility of GFAP and S100B protein in discriminating among MOGAD, AQP4-IgG NMOSD, and double seronegative patients was not consistent according to the few studies which tried to address this issue [80,82].

**Figure 1:**
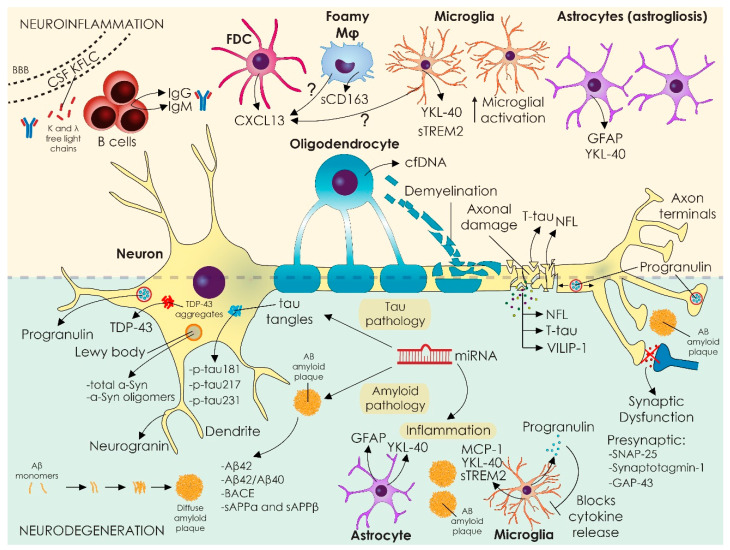
Novel and older classic biomarkers capturing various aspects of neuropathology in neuroinflammatory and neurodegenerative diseases. Neurodegeneration and inflammation are not always purely separated in diseases of CNS, and features of both processes appear in various disease settings with both qualitative and quantitative differences. Liquid-based biopsies in the CSF compartment that more closely resemble brain pathology, as it is in close proximity to the tissue, aim to unravel novel players in disease pathogenesis. In neuroinflammatory diseases such as MS, novel biomarkers reflect markers of oligodendrocyte death (circulating oligodendrocyte derived cell-free DNA), ongoing B cell activation and plasma cell maturation in the CNS (KFLC production, CXCL13 production), and overt microglial activation (YKL-40; sTREM2 release). In neurodegenerative diseases, various aggregated proteins (TDP43, tau, amyloid) characterize different clinical syndromes and can be captured with novel highly sensitive technical assays such as protein misfolding amplification assays and SiMoA. Moreover, low-grade inflammation, possibly initiated by misfolded and aggregated proteins (and vice-versa), is currently recognized, and known neuroinflammatory markers are also of clinical utility (YKL-40; s-TREM2, MCP-1). Other critical pathways in neurodegeneration, such as axonal damage and synaptic transmission compromise, are also captured by novel biomarkers such as neurofilaments and presynaptic proteins (SNAP25, GAP-43), respectively. Moreover, complex biomarkers such as progranulin, that apart from being a neuronal protein involved in neuritic growth, axonal transport, and synaptic function is also involved in controlling microglial activation, are becoming closer to clinical practice because they display abnormalities in core biological processes of relevant diseases. Cellular localization and cell of origin for displayed biomarkers can be visualized in the image. Not all tissue sources are displayed for simplicity reasons. Abbreviations: VLP-1; visinin-like protein 1, NF-L; neurofilament light, SNAP-25; synaptosomal associated protein 25, GAP-43; growth-associated protein 43, Ab1–42; the 42 amino acid form amyloid b, TDP-43; TAR DNA-binding protein 43, TREM2; Triggering receptor expressed on myeloid cells 2, YKL-40; also known as chitinase-3-like protein 1 (CHI3L1), a 40 kD chitin-binding protein with a YKL domain, MCP-1/CCL2; Monocyte chemoattract-ant protein-1, GFAP; glial fibrillary acidic protein, KFLC; CSF kappa free light chains, miRNA; micro-RNA, cf-DNA; cell-free DNA, CXCL13; chemokine (C-X-C motif) ligand 13, FDC; follicular dendritic cell, Aβ; amyloid-β, BACE; **beta-site amyloid precursor protein cleaving enzyme, sAPP; secreted amyloid precursor protein**.

The above-mentioned novel CSF biomarkers capturing inflammatory aspects either in autoimmune or neurodegenerative neurological diseases affecting CNS are summarized in Table 1 and depicted in Figure 1.

## 3. Biomarkers Tracking Neurodegenerative Aspects

### 3.1. Neurofilament Light Chain 

Neurofilaments are the major cytoskeletal proteins of neurons in both CNS and PNS, comprising light (NFL), medium, and heavy (NFH) neurofilament chains [87]. The most promising biomarker is light-chain NF, a native cytoskeletal protein, which is released into the CSF when axons are damaged, and thus it has been used as a biomarker of neuronal injury and neurodegeneration [88].

Intensive research over the past decades showed that in all clinical forms of MS, increased CSF NFL concentration reflects disease activity and progression [89]. CSF NFL levels correlate positively with serum levels, rendering blood an ideal mean for monitoring. Patients with relapse or with radiologic activity display significantly higher serum NFL levels than those in remission, and importantly, effective disease-modifying treatments, reduce NFL levels [90]. CSF NFL levels also have a predictive value for new or enlarging T2 lesions, brain volume loss, and risk of disability worsening [91]. CSF NFL levels and OBs were found to be independent risk factors for fostering the development of CIS and clinically definite MS in RIS syndrome [9].

Increased levels of NFL have been found in other neuroinflammatory conditions, such as MOGAD [92,93]. We recently showed that NFL is significantly increased in patients with paraneoplastic form autoimmune encephalitis. CSF-NFL levels with a cut-off value of 969 pg/mL had a sensitivity and specificity of 100% and 76.19%, respectively, regarding the detection of underlying malignancies [92,93].

Widespread axonal injury is a prominent feature of NMO pathophysiology, compared to MS, with optic nerves and spinal cord being severely affected. Initially, high CSF levels of NF heavy subunit (NFH) in NMO patients but not in MS patients had been shown [94,95]. Subsequent studies assessed CSF NF light subunit (NFL) levels in NMO patients which were found higher compared to MS, and other non-inflammatory neurological diseases patients. Importantly, NMO patients with increased CSF NFL levels during relapse displayed increased disability, a notion highlighting the role of NFL in capturing disease severity [96]. CSF NFL correlated with clinical and radiological aspects of disease severity, but were not able to discriminate NMO from MS [28,96].

Several studies have also found that CSF NFLs levels may be a marker of clinical severity of AD and MCI when compared to Aβ and tau proteins, reflecting future cognitive decline. Nevertheless, NFLs are not a specific marker for AD pathophysiology, as they are also found increased in other neurodegenerative diseases, while they normally also increase with age [97,98].

In movement disorders, such as PD-plus syndromes, NFLs show a good discriminatory power for the differentiation of PD, DLB, and PD dementia (PDD) but not multiple system atrophy (MSA). Scientific efforts for the inclusion of CSF NFL determination in the diagnostics of ALS are currently in progress [99,100,101,102]. Moreover, NFLs have been suggested as a prescreening tool for suspected CJD cases as an alternative to t-tau or 14-3-3 levels [103].

### 3.2. VILIP-1

VILIP-1, visinin-like protein 1, belongs to a family of proteins that are neuronal calcium sensors (NCS) and is highly expressed predominantly in neurons from pyramidal and non-pyramidal areas in AD brains [104]. VILIP-1 is associated with cardinal pathologic hallmarks of AD, as found placed close to dystrophic nerve cell processes, neuritic plaques, and fibrillar tangles [105]. The neurotoxic effects of VILIP-1 have been attributed to perturbations of Ca^2+^ homeostasis in AD and thus serve as a marker of neuronal injury [106]. Regarding CSF, elevated VILIP-1 levels have been reported in AD and MCI patients as compared to controls, while they were found to be strongly correlated with p-tau 181 and t-tau levels [107]. A recent meta-analysis showed that CSF VILIP-1 levels in AD are significantly higher compared to healthy individuals but not in those with MCI or DLB [108]. Furthermore, CSF VILIP-1 levels were significantly higher in patients with MCI who progressed to AD in comparison to those with stable MCI; however, the restricted number of studies does not allow clear conclusions. Interestingly, CSF levels of VILIP-1 are associated with the rate of brain atrophy in AD, while the ratio of VILIP-1/Aβ-42 significantly correlates with the brain amyloid load [109,110]. Therefore, VILIP-1 together with Aβ42 could be used for predicting the future rate of cognitive decline.

No solid evidence exists for the role of this biomarker in neuroinflammatory diseases.

### 3.3. Ubiquitin C-Terminal Hydrolase L1

CSF ubiquitin C-terminal hydrolase L1 (UCH-L1), a protein involved in the maintenance of axonal integrity, is involved in the pathway leading to the degradation of highly ubiquitinylated aggregated and/or damaged proteins by the 26S ubiquitin–proteasome system (UPS) [111,112]. UCH-L1 has been found to be increased predominantly in AD patients vs. controls or patients with other dementias and patients with MCI. It has been suggested that UCH-L1 might be implicated in AD pathophysiology through its interplay with the tau protein [113,114]. Towards this, CSF UCH-L1 positively correlated with CSF p-tau and neuron-specific enolase, a marker of neurodegeneration [115]. Moreover, a proteome analysis in CSF samples of ALS and FTD patients carrying *C9orf72* gene mutation showed that UCHL1 was among the most highly upregulated proteins in ALS patients [116]. No significant changes in CSF- UCH-L1 levels have been found in MS patients compared to controls [117].

## 4. Biomarkers Tracking Synaptic Pathology

### 4.1. Neurogranin

Neurogranin (Ng) is a calmodulin-binding postsynaptic protein playing a role in synaptic plasticity. Reduced protein expression of Ng has been described in the brains of AD patients but was found conversely increased in the CSF. The most important finding with clinical applications is high CSF levels of Ng reported in MCI patients progressing to AD compared to cognitively stable MCI patients and control individuals, making CSF Ng a surrogate biomarker for identifying patients in early disease stages [118,119,120]. Indeed, highly sensitive new ELISA assays have revealed high levels of Ng in CSF, in AD, and MCI-AD. CSF Ng levels predict conversion from MCI to AD and are associated with a faster rate of cognitive decline within amyloid-positive MCI patients [119]. A cut-off at 382 pg/mL of CSF Ng levels was found to correlate with the future rates of hippocampal atrophy as measured by MRI and rates of decrease in cortical glucose metabolism illustrated by FDG-PET. CSF Ng has also been correlated with many aspects of AD pathology, both with known biochemical changes in CSF t-tau and Aβ42 levels, as well as with tissue abnormalities such as the deposition of tau neurofibrillary deposits and β-amyloid plaques [121]. Moreover, other studies have shown that Ng could be a disease-specific biomarker as its levels were found to be elevated exclusively in patients with AD, but not in other neurodegenerative disorders, such as FTD, LBD, PD, progressive supranuclear palsy (PSP), or multiple system atrophy (MSA), with the only exception of a speech variant of FTD (svFTD = semantic variant of FTD) [120].

### 4.2. SNAP-25

SNAP-25 is a component of the SNARE complex, which is central to synaptic vesicle exocytosis, and by directly interacting with different calcium channels subunits, it negatively modulates neuronal voltage-gated calcium channels, thus regulating intracellular calcium dynamics. [122,123]. A recent study showed that CSF SNAP-25 concentrations were elevated in AD and CJD patients but not in other diseases such as PD spectrum, FTD, and ALS [124]. Moreover, SNAP-25 CSF levels correlated with higher Aβ load (as measured by CSF Aβ42/40 and Aβ PET Centiloid values) and were found higher in APOE ε4 carriers implying its role in relation to amyloid pathology in the AD continuum [125,126]. Another study found that CSF SNAP-25 levels were higher in patients suffering from MCI who were amyloid-β-positive compared to cognitively normal individuals (amyloid-β-positive or -negative). Therefore, the use of this marker in preclinical AD deserves further evaluation, whereas it could represent a useful diagnostic and prognostic biomarker for the earliest symptomatic stage of AD [127,128].

### 4.3. GAP-43

GAP-43 is a presynaptic protein expressed in various brain regions (hippocampus, entorhinal cortex, neocortex, and olfactory bulb) and is critically involved in synaptogenesis and neuronal plasticity in the adult brain [129]. CSF GAP-43 levels have been found to be increased in preclinical AD and, along with high CSF, Ng levels were associated with increased brain metabolism but lower cortical thickness in AD-related brain regions [126,130,131]. Moreover, high CSF GAP-43 levels were closely associated with MCI progression to dementia over a median of four years’ follow-up, while its levels correlated with CSF p-Tau181, suggesting a role of tau aggregations in presynaptic dysfunction [132]. CSF levels of GAP-43 have been measured in MS but with contradictory results [133,134].

The above-mentioned novel CSF biomarkers capturing neurodegenerative aspects as well as synaptic pathology either in autoimmune or neurodegenerative neurological diseases affecting CNS are summarized in Table 2 and depicted in Figure 1.

## 5. Biomarkers Tracking Disease-Specific Proteins

### 5.1. Alpha-Synuclein

Alpha-synuclein (α-Syn) is a neuronal protein that regulates synaptic vesicle trafficking and subsequent neurotransmitter release. It is the major constituent of Lewy bodies the pathogenic hallmark of PD and DLB, as well as in variable aggregated species in MSA, disorders collectively called synucleinopathies. A-Syn is readily secreted into extracellular space and can be found in different forms (monomeric, oligomeric, and seeding-competent aggregated forms) in CSF. Quantification of total α-Syn levels in CSF in multiple studies has shown a general trend of decreased α-Syn levels in patients with PD compared to healthy individuals. Interestingly, a recent study of our group showed that in general synucleinopathies exhibit lower total-α-Syn and higher phosphoS129-α-syn/total-α-Syn ratios compared to tauopathies [135].

A major limitation in measuring CSF α-Syn levels is the possible contamination by red blood cells, which is a big source of the protein and gives false-positive results [136]. The use of specific assays designed to amplify and detect very low amounts of aggregated α-Syn in biological samples, collectively termed as α-Syn seed amplification assays (SAAs), e.g., real-time quaking-induced conversion (RT-QuIC) and protein-misfolding cyclic amplification (PMCA), have promisingly shown the presence of seeding-competent α-Syn species in CSF, which could serve as a more accurate and reliable biomarker for PD and other synucleinopathies. Particularly high diagnostic performances of α-Syn SAAs measured in CSF, differentiating synucleinopathies from non-synuclein-related parkinsonism have been presented in different studies. PD could be discriminated from PSP, CBD with sensitivities that vary from 91 to 94% and sensitivities that reach 100% [137,138]. The discrimination between PD and MSA is more difficult as different conformational species of aggregated a-synuclein are involved in disease pathogenesis. Recently published studies have assessed the diagnostic value of CSF α-Syn seed quantification in diagnosis and differentiation among synucleinopathies. A-syn RT-QuIC could discriminate PD from MSA with a sensitivity of 75% based on quantitative characteristics of the assay (T50 and Vmax). Importantly, specific RT-QuIC parameters correlated with worse clinical progression of patients diagnosed with MSA but not PD [139].

### 5.2. TAR DNA-Binding Protein of 43kDa (TDP-43)

Frontotemporal lobar degenerations (FTLDs) comprise a spectrum of complex and heterogeneous neurodegenerative disorders characterized by degeneration of the frontal and anterior temporal lobes involving multiple clinical phenotypes, different protein aggregates (tau, TDP-43, fused in sarcoma protein-FUS) in tissue pathology and many genetic loci explaining up to 40% of familial FTLD [140]. The most intriguing aspect is that there is variability and overlapping in clinical, genetic, and histopathologic features, making it difficult to identify a unique biomarker signature [141,142]. TDP-43, encoded by the *TARDBP* gene, is the major aggregated protein involved in the formation of the characteristic inclusions, especially in its hyperphosphorylated and ubiquitin-bound form, in the brains of patients with ubiquitin-positive frontotemporal lobar degeneration (FTLD-U) and or ALS [141]. More than 50 mutations in the *TARDBP* gene have been identified across the FTD/ALS spectrum [143,144].

About 50% of FTLDs have positive TDP-43 aggregates, thus rendering TDP-43 an emerging disease-specific biological marker for the FTD/ALS spectrum [145]. Recently, it has been reported that TDP-43 levels in CSF were higher in patients with FTD and ALS than in controls, using Western blot along with chemiluminescence assays [146], while similar results were obtained by measuring TDP-43 in the CSF of patients with early-stage ALS using the ELISA technique [147,148]. Work from our lab has recently shown that CSF TDP-43 combined with tau proteins in the TDP-43 × t-tau/p-tau formula has good sensitivity and specificity for the discrimination of ALS-FTD spectrum disorders from controls [149,150,151].

Plasma levels of phosphorylated TDP-43 protein have been also associated with the presence of histopathological lesions of FTLD [152]. It seems, therefore, appropriate to intensify research efforts in broadening the classification system of dementias so that the diagnostic “arsenal” in the coming years will be enriched with further biological markers and the AT(N) system will evolve into ATT(N), where the second T corresponds to the TDP-43 proteinopathy [6].

### 5.3. Progranulin

The protein progranulin (PGRN) is the product of the *GRN* gene, a cysteine-rich protein that is glycosylated and secreted as a glycoprotein [153]. The structure of PGRN allows it to be broken down, by several proteases, into approximately seven ∼6 kDa granulins (GRNs) and a para-GRN of 3.5 kDa, consisting of 12 cysteine repeat motifs [154]. PGRN is a pleiotropic protein and the properties of the full-length protein are distinct from those of granulins. PGRN is considered to be a growth factor, especially for neurons, and important for their survival and outgrowth, whereas some GRNs have inflammatory properties [155]. Progranulin, which is also expressed in microglia, exerts a negative effect on neuroinflammatory processes, such as microgliosis and astrogliosis, and is also involved in repair mechanisms after axonal injury [156]. Moreover, PGRN also found in late endosomes and lysosomes, is associated with the maintenance of lysosome homeostasis, further supported by the finding that PGRN haploinsufficiency causes lysosome dysfunction [157,158,159].

Most attention has been given to PGRN due to the loss-of-function mutations in the progranulin gene (GRN) that have a pathogenic role in familial forms of FTD [160]. Pathogenic mutations of the *GRN* gene account for 20% of familial and 5% of sporadic cases of FTD, with variation in clinical expression, mainly expressed as primary progressive aphasia (PPA) and bvFTD (a behavioral variant of FTD), but rare phenotypes resembling AD and parkinsonism have also been reported [161,162]. At the neuropathology level, *GRN* mutations, in contrast to those of *MAPT* gene encoding tau, are associated with FTLD-TDP proteinopathy [163]. CSF progranulin has also been found to be decreased in the CSF and plasma of mutation carriers but is less extensively studied and a relatively weak correlation has been observed between CSF and blood [150,164,165]. CSF progranulin levels were found to be reduced not only to the rare cases of genetic GRN-FTD but also to the more common GRN-negative cases of FTD. Nevertheless, specific cut-offs are not available to precisely discriminate among carriers and non-carriers in order to avoid the high cost of genetic screening in FTD patients for the identification of GRN mutations [166].

Previous studies had reported no differences in CSF progranulin levels among AD, MCI, and controls [167]. Recently it was found that CSF levels of progranulin increase as early as ten years before the clinical presentation of the disease in patients with familial AD; thus, progranulin could represent a possible marker for early prediction of the disease onset. Higher CSF PGRN was linked to more advanced disease stages and cognitive decline in late-onset AD [51,155]. Nevertheless, it is not absolutely specific to AD and has been found to be deregulated in non-AD diseases as well.

As regards MS patients, CSF levels of PGRN had shown contradictory results. A recent study has unraveled minor only perturbations of this biomarker in CSF of patients with different clinical forms of MS [168]. Interestingly, RIS patients exhibited higher median CSF PGRN levels than healthy controls and showed no significant differences compared with CIS, RRMS, and PPMS cases [169,170]. The field of research in progranulin biology is promising, considering the ability of progranulin and/or granulins to capture treatment response in the era of trials of GRN-targeted therapies.

## 6. Molecular Biomarkers

### 6.1. MicroRNAs

MicroRNAs (miRNAs), are single-stranded 19–23 nucleotides long, nonprotein-coding RNA molecules that act as post-transcriptional regulators to fine-tune protein expression levels either by promoting mRNA degradation or by diminishing protein translation. miRNAs are secreted from cells to extracellular spaces through vehicle mediated (e.g., exosomes, microvesicles, apoptotic bodies) and not-vehicle-mediated pathways (bound with high-density lipoproteins and Ago2 proteins) [171]. A valuable source of circulating miRNAs is the CSF, and new studies are focusing on it for novel biomarkers discovery.

In MS, miRNAs could be a very useful and informative tool because it includes and transports material outside the cell originating from many cell types. This is the feature that renders miRNAs an important surrogate biomarker providing information on elusive cells and difficult-to-access tissues [172]. Various miRNAs have been recently described with perturbed expression in CSF of MS patients (miR-181c; miR-150; miR-328; miR-34c-5p; miR-142-3p; miR-let-7b-5p) with miR-181c and miR-150 to be associated with an earlier conversion of CIS to MS [173,174,175,176,177]. Importantly, a recent study showed that specific miRNA in CSF of MS patients could differentiate patients in remission from those in relapse. Additionally, they are associated with the extent of intrathecal inflammation, and they are involved in the cell cycle, immunoregulation, and neurogenesis [178]. The implication of CSF miRNAs in important aspects of MS pathophysiology and especially during relapse stages was also supported by another study that found that the immune-related pathways controlled by these differentially expressed miRNAs are involved in the activation of T and B cells, as well as cytokine and chemokine signaling such as transforming growth factor beta (TGF-β) [179].

Regarding AD pathogenesis, miRNAs have been shown to target molecular pathways associated with pathologic processes that are implicated in disease evolution, such as synaptic and mitochondrial dysfunction, Aβ accumulation, and tau toxicity. In 2013 Sala Frigerio et al. [180] by applying qRT-PCR (real-time quantitative PCR) in CSF samples showed diminished levels of miR-27a-3p in AD patients, which were associated with classical CSF AD-related biomarkers levels of tau and β-amyloid). Burgos et al. [181], by using next-generation sequencing, profiled the miRNA content from 69 patients with Alzheimer’s disease, 67 with Parkinson’s disease, and 78 neurologically controls in both serum and CSF. Importantly, they noticed that miRNAs present in the CSF differentiate patients quite more effectively than miRNAs in serum. Specifically, miR-101 was found to be reduced in CSF and correlated with the presence of neurofibrillary tangles and plaque density. miRNA-9 was also downregulated in CSF from AD patients, and its expression levels changed dynamically across Braak stages. Kiko et al. [182] indicated that miR-29a and miR-29b levels were higher, and miR- 34a, miR-125b, and miR-146a levels were lowered in the CSF samples of AD patients. Among the various miRNAs studied by Muller et al. 2016, only miR-29a increased by a factor of 2.2 in CSF samples of AD patients [183]. Finally, miR-29c-3p, miR15a-5p, and let-7i-5p were found as three differential expressed miRNAs in CSF of AD patients that could all be related to AD relevant targets such as APP (amyloid precursor protein) and BACE1 (Beta-secretase 1), implying that miRNA is actively involved in key pathogenetic AD processes [184].

Apart from AD, the application of miRNA as a potential biomarker has been tested in many other neurodegenerative diseases. miR-9-3p and miR-106b-5p levels in CSF have been found capable of discriminating PD from MSA patients with good diagnostic accuracy by receiver operating characteristics curve evaluation (area under the curve = 0.73). In the same study, a single microRNA, miR-106b-5p, provided the best discrimination between PD and PSP (area under the curve = 0.85) in the CSF [185].

De Felice et al. suggested that miR-338-3P, which is found in both blood and CSF, could be used as a biomarker for patients with ALS. They have shown that miR-338-3p is highly expressed in ALS tissues by in situ hybridization staining and that was specifically localized in the grey matter of spinal cord tissues from sALS autopsied patients [186].

The limitations in the wide use of miRNAs mainly rely on the high cost of the techniques applied for their measurement, the need for bioinformatic tools for analyzing complex networks in which they are involved, the variability in levels (depending on gender, aging, comorbidities), and the need for universal clear-cut off levels that require standardized protocols.

### 6.2. Cell-Free DNA (Genomic and Mitochondrial Origin)

Cell-free DNA (cfDNA) constitutes externalized, short, fragmented DNA in various lengths found in bodily fluids and is the product of either programmed cell death, necrosis, or cell activation [187]. A breakthrough in the so-called “liquid biopsy” research area is the identification of CSF-derived cell-free DNA that captures mosaic somatic mutations in malignant brain tumors [188,189,190]. Regarding non-malignant neurodegenerative diseases, somatic mutations have been recently described in AD brain specimens and were found enriched in PI3K-AKT, MAPK, and AMPK pathway genes known to contribute to hyperphosphorylation of tau. Importantly, pathogenic brain somatic mutation in PIN1 leads to a loss-of-function mutation. Nevertheless, the use of these genetic alterations as diagnostic biomarkers has not been assessed in bigger cohorts and has not been validated yet.

The role of cfDNA in AD has emerged in the last years. DNA methylation has been shown to be altered in various tissues and brain areas of AD patients. Other genes have been found hypermethylated (APP, trem2, ank1) and other hypomethylated (PINI1) [191]. Highly methylated neuronal tissue-specific LHX2 gene was found to be increased in the plasma cfDNA of patients with AD, particularly at the early stage of the disease [192]. Recently, significant genome-wide methylation changes in circulating cfDNA from AD subjects along with artificial intelligence platforms revealed deregulated methylation profiles in genes epigenetically altered in AD associated with synaptic activity, neuronal stemness, and age-dependent neurodegeneration [193].

Methylation analysis patterns in cfDNA have gained much attention as they provide information on the tissue of origin. In a pioneer study by Lehmann-Werman and colleagues in 2016, oligodendrocyte-derived DNA was found to be enriched in the cell-free DNA of patients with relapsing MS [194]. Additionally, demethylated MOG cfDNA could serve as a biomarker of oligodendrocyte death and was found higher in active disease compared to those with inactive and healthy controls. So, the identification of specific methylation patterns in the peripheral blood of cells critically involved in brain pathology could represent a promising tool for the diagnosis and monitoring of neuroinflammatory diseases.

Perturbations in the methylation status of various genes extend beyond AD. A differentially methylated region located in the promoter–enhancer region of the rhomboid 5 homolog 2 (RHBDF2) gene was identified in ALS patients in cfDNA in the plasma [195]. Overall, cfDNA levels were recently found increased in ALS patients. Caggiano et al. [196] used CelFiE in the cfDNA samples and found expanded skeletal muscle-derived DNA in patients with ALS. Nevertheless, studies on CSF are lacking and it is currently unknown their correlation with disease progression or severity or with genetic variants of the disease. Collectively, the utility of CSF methylome deconvolution in studying human tissue dynamics in neurological disorders adds insights beyond the use of cfDNA as a biomarker but points to its role as a hallmark of the underlining pathology. While these methylation marks may be a biomarker of disease regardless of their origin, the use of brain-derived cfDNA to identify new epigenetic biomarkers is relevant because these marks may reflect more accurately the molecular and epigenetic changes that are occurring in the damaged brain tissue.

The release of DNA of mitochondrial origin (cf-mtDNA) in the CSF has been studied more extensively than cfDNA. mtDNA that have been misplaced into the cytosol or released into the bloodstream holds inflammatory properties as it could serve as damage-associated molecular patterns activating cytosolic receptors and TLRs [197]. Again, in the settings of MS, assessment of cf-mtDNA has resulted in opposite results. Reduced cf-mtDNA was found in progressive MS and is considered a hallmark of broader neurodegeneration [198]. On the other hand, increased cf-mtDNA, measured with digital droplet PCR in CSF, was found in patients with progressive MS compared to non-inflammatory neurologic disease controls. Importantly, higher T2 lesion volumes and lower normalized brain volumes were associated with increased concentration of mtDNA. Moreover, cf-mtDNA was amenable to therapeutic intervention as patients treated with fingolimod had significantly lower mtDNA copy levels at follow-up [199].

Studies assessing the application of cf-mtDNA as a reliable biomarker in AD have revealed contradictory results. For example, reports of a significant decrease in CSF cf-mtDNA levels in AD patients have not been replicated or even resulted in opposite results [200]. One group, by applying droplet digital polymerase chain reaction in CSF specimens, described significantly higher CSF mtDNA copies/μL compared to neurologically healthy controls, but not with a very good discriminative capability (in the receiver-operating characteristic analysis, area under the curve of 0.715 for distinguishing AD patients from controls). In other neurodegenerative diseases and specifically in Parkinson’s disease, cf-mtDNA was found significantly decreased in the CSF of patients and the reduction was associated with the type and time length of treatment [201]. Nevertheless, it was affected by comorbidities, a notion that limits its use as a disease-specific biomarker [202]. Molecular biomarkers identified in liquid biopsies from patients with neurodegenerative and autoimmune inflammatory disorders affecting CNS are presented in Table 3.

## 7. Future Directions

The most critical step for future biomarkers discoveries is understanding disease-related pathogenetic mechanisms and unraveling the disturbed architecture of the tissue, cells, and the corresponding implicated biological pathways. An overall picture of the localization and distribution of all novel biomarkers in CNS, presented in this review, is illustrated in Figure 1.

Established and novel biomarker for use in clinical practice for diagnosis, prognosis, and monitoring disease evolution, especially in the era of new drug discovery imposes the need for sensitive and accurate analytical methods producing homogeneous quantification results, such as those produced on fully automated laboratory instruments.

Standardization among platforms, improvement of coefficients of variation, and the use of internal and external quality control programs are necessary both in clinical and research settings.

Recent advances in methodologies have brought novel research tools, such as ultrasensitive immune-based technologies, such as SiMoA, capable of quantifying proteins at very low concentrations, and mass spectrometry-based proteomics both bearing promises for the discovery of new CSF biomarkers, tracking neuroinflammation and neurodegeneration aspects. In addition, novel “seeding” assays are under development, which could be used for amplification and capture of the abnormally aggregated misfolded proteins with prion-like properties, hallmarks of neurodegenerative diseases.

Finally, novel molecular biomarkers are to be recognized with molecular techniques such as new-generation RNA sequencing, found at both cell-free and cell-rich compartments of CSF. Future delegate CSF DNA studies of methylation analysis or whole sequencing analysis will pave the way for new molecular and genetic/epigenetic markers to be identified thus, expanding the discovery of biomarkers to the nucleome.

## 8. Conclusions

After more than a century following the introduction of lumbar puncture in medical practice, CSF laboratory analysis has never stopped being used as a powerful diagnostic tool mainly for infectious and autoimmune/demyelinating diseases of the CNS. The introduction of biomarkers for neurodegeneration in recent years marks another milestone in CSF diagnostics. Nowadays, there is an urgent need to open the field of biomarkers research beyond those already established. Under this perspective, we have tried to provide new evidence on novel biomarkers either for autoimmune inflammatory or neurodegenerative disorders.

Studies have shown that single biomarker measurements are not able to capture disease pathobiology as a whole, while many biomarkers are not disease-specific but common in both neurodegenerative and neuroinflammatory diseases. Thus, future biomarker combination approach(es) (in the best possible scheme) integrating both old and new ones would permit proper identification of disease-specific mechanisms and contribute to a more precise diagnosis and subsequent treatment, while facilitating the enrollment of biologically homogeneous cohorts of patients in clinical trials.

## Figures and Tables

**Table 1:** CSF-based biomarkers tracking neuroinflammation.

Biomarker	Specific Biological Process	Neurodegenerative Diseases	Neuroinflammatory Diseases	Methodology (Most Used)
KFLC	Intrathecal immunoglobulin synthesis	N/A	MS, CIS	Nephelometry
YKL-40	Glial activation	AD, ALS, CJD, FTD	RRMS, CIS, NMO	Elisa
sTREM2	Glial activation	AD, PD, FTD, CJD	MS	Elisa
sCD163	Activated microglia and tissue macrophages	Not studied	MS	Elisa
CXCL13	B-cell activation and recruitment to CNS	Not studied	RRMS, CIS, NMO	Elisa
IL-6	Th2 and Th17-related inflammation	Not studied	NMO	Elisa
MCP-1/CCL2	Glial activation, monocyte recruitment	AD	MS (reduced)	Elisa
GFAP	Glial activation (astrocytes)	AD	SPMS > RPMS, NMO	Elisa

Abbreviations: KFLC: kappa free light chains, YKL-40: also known as chitinase-3-like protein 1 (CHI3L1): a 40 kD chitin binding protein with a YKL domain. TREM2: triggering receptor expressed on myeloid cells 2, sCD163; soluble cluster of differentiation 163, CXCL13; chemokine (C-X-C motif) ligand 13, IL-6; interleuκin 6, MCP-1/CCL2; Monocyte chemoattractant protein-1, GFAP; Glial fibrillary acidic protein, AD; Alzheimer’s disease, FTD, frontotemporal dementia, PD; Parkinson’s disease, CJD; Creutzfeldt–Jakob disease, MS; multiple sclerosis, SPMS; secondary progressive multiple sclerosis; RRMS; relapsing-remitting multiple sclerosis, CIS; clinically isolated syndrome, NMO; neuromyelitis optica.

**Table 2:** CSF-based biomarkers tracking neurodegeneration and synaptic pathology.

Biomarker	Specific Biological Process	Neurodegenerative Diseases	Neuroinflammatory Diseases	Methodology (Most Used)
NF-L	Axonal dysfunction	AD, FTD, VaD, CJD, ALS, PSP, MSA, CBD	RRMS, SPMS, PPMS, CIS, NMO, MOGAD	ELISA
VILIP-1	Axonal dysfunction	AD	Not studied	ELISA
UCH-L1	Axonal dysfunction	AD	No difference	ELISA
Neurogranin	Synaptic degeneration	AD	Not studied	ELISA, mass spectrometry
SNAP-25	Synaptic degeneration	AD	not studied	ELISA, mass spectrometry, SiMoA
GAP-43	Synaptic degeneration	AD	contradictory results	ELISA

Abbreviations: NF-L: neurofilament light, VILP-1: visinin-like protein 1, UCH-L1: ubiquitin C-terminal hydrolase L1; SNAP-25; synaptosomal associated protein 25, GAP-43; growth-associated protein 43, AD; Alzheimer’s disease, FTD; frontotemporal dementia, PD; Parkinson’s disease; VaD; vascular dementia; CJD; Creutzfeldt–Jakob disease, PSP; progressive supranuclear palsy, MSA; multiple system atrophy, CBD; corticobasal degeneration, DLB; dementia with Lewy bodies, ALS; amyotrophic lateral sclerosis, MS; multiple sclerosis, SPMS; secondary progressive multiple sclerosis, RRMS; relapsing-remitting multiple sclerosis, PPMS; primary progressive MS, CIS; clinically isolated syndrome, NMO; neuromyelitis optica, MOGAD; anti-myelin oligodendrocyte glycoprotein antibodies associated disorders, SiMoA; single molecule array.

**Table 3:** CSF-based biomarkers tracking protein pathologies and novel molecular biomarkers.

Protein Metabolism/Aggregation				
Biomarker	Specific Biological Process	Neurodegenerative Diseases	Neuroinflammatory Diseases	Methodology (Most Used)
a-synuclein	a-Syn pathology	AD, PD, CBD, MSA, DLB	Not applicable	ELISA, RT-QuIC, PMCA
TDP-43	TDP-43 metabolism	ALS, FTD	Not applicable	ELISA
Progranulin	Glial activation inhibitor	AD, FTD	MS	ELISA
**Molecular biomarkers**				
miRNA	Various molecular targets implicated in molecular networks	AD (miR-27a-3p miR-101 miR-29a, miR-29b, miR- 34a, miR-125b, miR-29c-3p, miR15a-5p, let-7i-5p, miR-146a) PD (miR-7-5p, miR-331-5p, miR-145-5p), MSA (miR-9-3p and miR-106b-5p), PSP (miR-106b-5p), ALS (miR-338-3P)	MS (miR-181c; miR-150; miR-328; miR-34c-5p; miR-142-3p; miR-let-7b-5p and miR-15a-3p/124-5p/149-3p/29c-3p/33a-3p/34c-5p/297)	RT-qPCR, NGS
Cell-free DNA and mitochondrial DNA	Cell death	AD, PD	MS	RT-qPCR, ddPCR, fluorometric analysis, sequencing for mutations, methylation sequencing (qMSP)

Abbreviations: TDP-43: TAR DNA-binding protein 43, FTD: frontotemporal dementia; AD: Alzheimer’s disease; PD: Parkinson’s disease, VaD: vascular dementia, CJD: Creutzfeldt–Jakob disease, PSP: progressive supranuclear palsy, MSA: multiple system atrophy, CBD: corticobasal degeneration, DLB: dementia with Lewy bodies, ALS: amyotrophic lateral sclerosis, MS: multiple sclerosis, SPMS: secondary progressive multiple sclerosis, RRMS: relapsing-remitting multiple sclerosis, CIS: clinically isolated syndrome, miRNA: micro-RNA, qMSP: quantitative methylation-specific PCR, RT-qPCR: reverse transcriptase quantitative polymerase chain reaction, ddPCR: droplet digital polymerase chain reaction, NGS; next-generation sequencing, PMCA; protein misfolding cyclic amplification, RT-QuIC; real-time quaking-induced conversion.

## Data Availability

Not applicable.

## Abbreviations

Abs	autoantibodies
AD	Alzheimer’s disease
ALS	amyotrophic lateral sclerosis
APP	Amyloid precurcor protein
AQP4	aquaporin-4
α-Syn	alpha-synuclein
BACE1	Beta-secretase 1, also known as beta-site amyloid precursor protein cleaving enzyme 1
BBB	blood–brain barrier
bvFTD	behavioral variant of FTD
CBD	corticobasal degeneration
cfDNA	cell-free DNA
cf-mtDNA	mitochondrial cell-free DNA
CHMP2B	charged multivesicular body protein 2B
CIS	clinically isolated syndrome
CJD	Creutzfeldt–Jacob disease
CSF	cerebrospinal fluid
CXCL13	chemokine (C-X-C motif) ligand 13
ddPCR	droplet digital polymerase chain reaction
DLB	Lewy bodies
DMT	disease-modifying therapies
EDSS	Expanded Disability Status Scale
ELISA	enzyme-linked immunosorbent assay
FDG-PET	fluorodeoxyglucose PET
FLC	free light chains
FTD	frontotemporal dementia
FTLDs	frontotemporal lobar degenerations
FTLD-U	ubiquitin-positive frontotemporal lobar degeneration
FUS	fused in sarcoma protein
GAP-43	growth-associated protein 43
GFAP	glial fibrillary acidic protein
GRNL	granulin
IL-13	interleukin-13
IL-17	interleukin-17
IL-5	interleukin-5
IL-6	interleukin-6
IL-8	interleukin-8
KFLC	kappa free light chain
KCSF	kappa free light chain of cerebrospinal fluid
LFLC	lambda free light chain
MCI	mild cognitive impairment
MCP-1	monocyte chemoattractant protein-1
miRNAs	microRNAs
MOGAD	myelin oligodendrocyte glycoprotein antibody disease
MRI	magnetic resonance imaging
MS	multiple sclerosis
MSA	multiple system atrophy
NAWM	normal-appearing white matter
NDDs	neurodegenerative diseases
NF	neurofilaments
NFH	heavy neurofilament chains
NFL	neurofilament light protein
Ng	neurogranin
NGS	next-generation sequencing
NIDs	neuroinflammatory diseases
NMOSD	NMO spectrum disorders
NMO	neuromyelitis optica
OBs	oligoclonal bands
PD	Parkinson’s disease
PDD	PD dementia
PET	emission tomography
PGRN	progranulin
PMCA	protein-misfolding cyclic amplification
PPA	primary progressive aphasia
PSP	progressive supranuclear palsy
p-tau	phospho-tau protein
qMSP	quantitative methylation-specific PCR
qRT-PCR	real-time quantitative PCR
RIS	radiologically isolated syndrome
RRMS	relapsing-remitting MS
RT-QuIC	real-time quaking-induced conversion
SAAs	seed amplification assays
sCD163	soluble cluster of differentiation 163
SiMoA	single molecule array
SNAP-25	synaptosomal associated protein 25
SPMS	secondary progressive MS
sTREM2	soluble TREM2
svFTD	semantic variant of FTD
TDP-43	TAR DNA-binding protein of 43 kDa
TGF-β	transforming growth factor beta
Th17	T helper 17 cells
Th2	T helper 2 cells
TREM2	triggering receptor expressed on myeloid cells 2
t-tau	total tau
UCH-L	ubiquitin C-terminal hydrolase L1
UPS	ubiquitin proteasome system
VaD	vascular dementia
VILIP-1	visinin-like protein 1
YKL-40	also known as chitinase-3-like protein 1 (CHI3L1), a 40 kD chitin binding protein with a YKL domain

## References

[1] Gaetani L., Paolini Paoletti F., Bellomo G., Mancini A., Simoni S., Di Filippo M., Parnetti L. (2020). CSF and Blood Biomarkers in Neuroinflammatory and Neurodegenerative Diseases: Implications for Treatment. Trends Pharmacol. Sci..

[2] Thompson A.J., Banwell B.L., Barkhof F., Carroll W.M., Coetzee T., Comi G., Correale J., Fazekas F., Filippi M., Freedman M.S. (2018). Diagnosis of multiple sclerosis: 2017 revisions of the McDonald criteria. Lancet Neurol..

[3] Graus F., Titulaer M.J., Balu R., Benseler S., Bien C.G., Cellucci T., Cortese I., Dale R.C., Gelfand J.M., Geschwind M. (2016). A clinical approach to diagnosis of autoimmune encephalitis. Lancet Neurol..

[4] Hampel H., Hardy J., Blennow K., Chen C., Perry G., Kim S.H., Villemagne V.L., Aisen P., Vendruscolo M., Iwatsubo T. (2021). The Amyloid-β Pathway in Alzheimer’s Disease. Mol. Psychiatry.

[5] Skillbäck T., Farahmand B.Y., Rosén C., Mattsson N., Nägga K., Kilander L., Religa D., Wimo A., Winblad B., Schott J.M. (2015). Cerebrospinal fluid tau and amyloid-β1-42 in patients with dementia. Brain.

[6] Jack C.R., Bennett D.A., Blennow K., Carrillo M.C., Dunn B., Haeberlein S.B., Holtzman D.M., Jagust W., Jessen F., Karlawish J. (2018). NIA-AA Research Framework: Toward a biological definition of Alzheimer’s disease. Alzheimer’s Dement..

[7] Michel L., Touil H., Pikor N.B., Gommerman J.L., Prat A., Bar-Or A. (2015). B Cells in the Multiple Sclerosis Central Nervous System: Trafficking and Contribution to CNS-Compartmentalized Inflammation. Front. Immunol..

[8] Pryce G., Baker D. (2018). Oligoclonal bands in multiple sclerosis; Functional significance and therapeutic implications. Does the specificity matter. Mult. Scler. Relat. Disord..

[9] Matute-Blanch C., Villar L.M., Álvarez-Cermeño J.C., Rejdak K., Evdoshenko E., Makshakov G., Nazarov V., Lapin S., Midaglia L., Vidal-Jordana A. (2018). Neurofilament light chain and oligoclonal bands are prognostic biomarkers in radiologically isolated syndrome. Brain.

[10] Ramsden D.B. (2017). Multiple sclerosis: Assay of free immunoglobulin light chains. Ann. Clin. Biochem..

[11] Rudick R.A., Peter D.R., Bidlack J.M., Knutson D.W. (1985). Multiple sclerosis: Free light chains in cerebrospinal fluid. Neurology.

[12] Bracco F., Gallo P., Menna R., Battistin L., Tavolato B. (1987). Free light chains in the CSF in multiple sclerosis. J. Neurol..

[13] Presslauer S., Milosavljevic D., Brücke T., Bayer P., Hübl W. (2008). Elevated levels of kappa free light chains in CSF support the diagnosis of multiple sclerosis. J. Neurol..

[14] Duranti F., Pieri M., Centonze D., Buttari F., Bernardini S., Dessi M. (2013). Determination of κFLC and κ Index in cerebrospinal fluid: A valid alternative to assess intrathecal immunoglobulin synthesis. J. Neuroimmunol..

[15] Hassan-Smith G., Durant L., Tsentemeidou A., Assi L.K., Faint J.M., Kalra S., Douglas M.R., Curnow S.J. (2014). High sensitivity and specificity of elevated cerebrospinal fluid kappa free light chains in suspected multiple sclerosis. J. Neuroimmunol..

[16] Makshakov G., Nazarov V., Kochetova O., Surkova E., Lapin S., Evdoshenko E. (2015). Diagnostic and Prognostic Value of the Cerebrospinal Fluid Concentration of Immunoglobulin Free Light Chains in Clinically Isolated Syndrome with Conversion to Multiple Sclerosis. PLoS ONE.

[17] Saadeh R.S., Bryant S.C., McKeon A., Weinshenker B., Murray D.L., Pittock S.J., Willrich M.A.V. (2022). CSF Kappa Free Light Chains: Cutoff Validation for Diagnosing Multiple Sclerosis. Mayo Clin. Proc..

[18] Leurs C.E., Twaalfhoven H., Lissenberg-Witte B.I., van Pesch V., Dujmovic I., Drulovic J., Castellazzi M., Bellini T., Pugliatti M., Kuhle J. (2020). Kappa free light chains is a valid tool in the diagnostics of MS: A large multicenter study. Mult. Scler..

[19] Christiansen M., Gjelstrup M.C., Stilund M., Christensen T., Petersen T., Jon Møller H. (2018). Cerebrospinal fluid free kappa light chains and kappa index perform equal to oligoclonal bands in the diagnosis of multiple sclerosis. Clin. Chem. Lab. Med..

[20] Zeman D., Kušnierová P., Bartoš V., Hradílek P., Kurková B., Zapletalová O. (2016). Quantitation of free light chains in the cerebrospinal fluid reliably predicts their intrathecal synthesis. Ann. Clin. Biochem..

[21] Gurtner K.M., Shosha E., Bryant S.C., Andreguetto B.D., Murray D.L., Pittock S.J., Willrich M.A.V. (2018). CSF free light chain identification of demyelinating disease: Comparison with oligoclonal banding and other CSF indexes. Clin. Chem. Lab. Med..

[22] Süße M., Hannich M., Petersmann A., Zylla S., Pietzner M., Nauck M., Dressel A. (2018). Kappa free light chains in cerebrospinal fluid to identify patients with oligoclonal bands. Eur. J. Neurol..

[23] Presslauer S., Milosavljevic D., Huebl W., Parigger S., Schneider-Koch G., Bruecke T. (2014). Kappa free light chains: Diagnostic and prognostic relevance in MS and CIS. PLoS ONE.

[24] Senel M., Mojib-Yezdani F., Braisch U., Bachhuber F., Lewerenz J., Ludolph A.C., Otto M., Tumani H. (2019). CSF Free Light Chains as a Marker of Intrathecal Immunoglobulin Synthesis in Multiple Sclerosis: A Blood-CSF Barrier Related Evaluation in a Large Cohort. Front. Immunol..

[25] Reiber H., Zeman D., Kušnierová P., Mundwiler E., Bernasconi L. (2019). Diagnostic relevance of free light chains in cerebrospinal fluid—The hyperbolic reference range for reliable data interpretation in quotient diagrams. Clin. Chim. Acta.

[26] Kirkpatrick R.B., Emery J.G., Connor J.R., Dodds R., Lysko P.G., Rosenberg M. (1997). Induction and expression of human cartilage glycoprotein 39 in rheumatoid inflammatory and peripheral blood monocyte-derived macrophages. Exp. Cell Res..

[27] Bonneh-Barkay D., Wang G., Starkey A., Hamilton R.L., Wiley C.A. (2010). In vivo CHI3L1 (YKL-40) expression in astrocytes in acute and chronic neurological diseases. J. Neuroinflamm..

[28] Cubas-Núñez L., Gil-Perotín S., Castillo-Villalba J., López V., Solís Tarazona L., Gasqué-Rubio R., Carratalá-Boscá S., Alcalá-Vicente C., Pérez-Miralles F., Lassmann H. (2021). Potential Role of CHI3L1+ Astrocytes in Progression in MS. Neurol. Neuroimmunol. Neuroinflamm..

[29] Hinsinger G., Galéotti N., Nabholz N., Urbach S., Rigau V., Demattei C., Lehmann S., Camu W., Labauge P., Castelnovo G. (2015). Chitinase 3-like proteins as diagnostic and prognostic biomarkers of multiple sclerosis. Mult. Scler..

[30] Comabella M., Fernández M., Martin R., Rivera-Vallvé S., Borrás E., Chiva C., Julià E., Rovira A., Cantó E., Alvarez-Cermeño J.C. (2010). Cerebrospinal fluid chitinase 3-like 1 levels are associated with conversion to multiple sclerosis. Brain.

[31] Schneider R., Bellenberg B., Gisevius B., Hirschberg S., Sankowski R., Prinz M., Gold R., Lukas C., Aiden Haghikia A. (2021). Chitinase 3-like 1 and neurofilament light chain in CSF and CNS atrophy in MS. Neurol. Neuroimmunol. Neuroinflamm..

[32] Francisco Pérez-Miralles F., Prefasi D., García-Merino A., Gascón-Giménez F., Medrano N., Castillo-Villalba J., Cubas L., Alcalá C., Gil-Perotín S., Gómez-Ballesteros R. (2020). CSF Chitinase 3-like-1 association with disability of primary progressive MS. Neurol. Neuroimmunol. Neuroinflamm..

[33] Condello C., Yuan P., Schain A., Grutzendler J. (2015). Microglia constitute a barrier that prevents neurotoxic protofibrillar Aβ42 hotspots around plaques. Nat. Commun..

[34] Antonell A., Mansilla A., Rami L., Lladó A., Iranzo A., Olives J., Balasa M., Sánchez-Valle R., Molinuevo J.L. (2014). Cerebrospinal fluid level of YKL-40 protein in preclinical and prodromal Alzheimer’s disease. J. Alzheimer’s Dis..

[35] Wang L., Gao T., Cai T., Li K., Zheng P., Liu J., Alzheimer’s Disease Neuroimaging Initiative (2020). Cerebrospinal fluid levels of YKL-40 in prodromal Alzheimer’s disease. Neurosci. Lett..

[36] Sutphen C.L., Jasielec M.S., Shah A.R., Macy E.M., Xiong C., Vlassenko A.G., Benzinger T.L., Stoops E.E., Vanderstichele H.M., Brix B. (2015). Longitudinal Cerebrospinal Fluid Biomarker Changes in Preclinical Alzheimer Disease During Middle Age. JAMA Neurol..

[37] Olsson B., Hertze J., Lautner R., Zetterberg H., Nägga K., Höglund K., Basun H., Annas P., Lannfelt L., Andreasen N. (2013). Microglial markers are elevated in the prodromal phase of Alzheimer’s disease and vascular dementia. J. Alzheimer’s Dis..

[38] Oeckl P., Weydt P., Steinacker P., Anderl-Straub S., Nordin F., Volk A.E., Diehl-Schmid J., Andersen P.M., Kornhuber J., Danek A. (2019). Different neuroinflammatory profile in amyotrophic lateral sclerosis and frontotemporal dementia is linked to the clinical phase. J. Neurol. Neurosurg. Psychiatry.

[39] Llorens F., Thüne K., Tahir W., Kanata E., Diaz-Lucena D., Xanthopoulos K., Kovatsi E., Pleschka C., Garcia-Esparcia P., Schmitz M. (2017). YKL-40 in the brain and cerebrospinal fluid of neurodegenerative dementias. Mol. Neurodegener.

[40] Piccio L., Buonsanti C., Cella M., Tassi I., Schmidt R.E., Fenoglio C., Rinker J., Naismith R.T., Panina-Bordignon P., Passini N. (2008). Identification of soluble TREM-2 in the cerebrospinal fluid and its association with multiple sclerosis and CNS inflammation. Brain.

[41] Azzolini F., Gilio L., Pavone L., Iezzi E., Dolcetti E., Bruno A., Buttari F., Musella A., Mandolesi G., Guadalupi L. (2022). Neuroinflammation is associated with GFAP and sTREM2 levels in multiple sclerosis. Biomolecules.

[42] Ohrfelt A., Axelsson M., Malmestrom C., Novakova L., Heslegrave A., Blennow K., Lycke J., Zetterberg H. (2016). Soluble TREM-2 in cerebrospinal fluid from patients with multiple sclerosis treated with natalizumab or mitoxantrone. Mult. Scler..

[43] Guerreiro R., Wojtas A., Bras J., Carrasquillo M., Rogaeva E., Majounie E., Cruchaga C., Sassi C., Kauwe J.S., Younkin S. (2013). TREM2 variants in Alzheimer’s disease. N. Engl. J. Med..

[44] Jonsson T., Stefansson H., Steinberg S., Jonsdottir I., Jonsson P.V., Snaedal J., Bjornsson S., Huttenlocher J., Levey A.I., Lah J.J. (2013). Variant of TREM2 associated with the risk of Alzheimer’s disease. N. Engl. J. Med..

[45] Suárez-Calvet M., Kleinberger G., Araque Caballero M.Á., Brendel M., Rominger A., Alcolea D., Fortea J., Lleó A., Blesa R., Gispert J.D. (2016). sTREM2 cerebrospinal fluid levels are a potential biomarker for microglia activity in early-stage Alzheimer’s disease and associate with neuronal injury markers. EMBO Mol. Med..

[46] Suárez-Calvet M., Araque Caballero M.Á., Kleinberger G., Bateman R.J., Fagan A.M., Morris J.C., Levin J., Danek A., Ewers M., Haass C. (2016). Early changes in CSF sTREM2 in dominantly inherited Alzheimer’s disease occur after amyloid deposition and neuronal injury. Sci. Transl. Med..

[47] Ewers M., Franzmeier N., Suárez-Calvet M., Morenas-Rodriguez E., Caballero M.A.A., Kleinberger G., Piccio L., Cruchaga C., Deming Y., Dichgans M. (2019). Alzheimer’s Disease Neuroimaging Initiative. Increased soluble TREM2 in cerebrospinal fluid is associated with reduced cognitive and clinical decline in Alzheimer’s disease. Sci. Transl. Med..

[48] Heslegrave A., Heywood W., Paterson R., Magdalinou N., Svensson J., Johansson P., Öhrfelt A., Blennow K., Hardy J., Schott J. (2016). Increased cerebrospinal fluid soluble TREM2 concentration in Alzheimer’s disease. Mol. Neurodegener..

[49] Henjum K., Almdahl I.S., Årskog V., Minthon L., Hansson O., Fladby T., Nilsson L.N.G. (2016). Cerebrospinal fluid soluble TREM2 in aging and Alzheimer’s disease. Alzheimer’s Res. Ther..

[50] Knapskog A.B., Henjum K., Idland A.V., Eldholm R.S., Persson K., Saltvedt I., Watne L.O., Engedal K., Nilsson L.N.G. (2020). Cerebrospinal fluid sTREM2 in Alzheimer’s disease: Comparisons between clinical presentation and AT classification. Sci. Rep..

[51] Suárez-Calvet M., Morenas Rodríguez E., Kleinberger G., Schlepckow K., Caballero M.Á.A., Franzmeier N., Capell A., Fellerer K., Nuscher B., Eren E. (2019). Early increase of CSF sTREM2 in Alzheimer’s disease is associated with tau related neurodegeneration but not with amyloid-β pathology. Mol. Neurodegener..

[52] Pascoal T.A., Benedet A.L., Ashton N.J., Kang M.S., Therriault J., Chamoun M., Savard M., Lussier F.Z., Tissot C., Karikari T.K. (2021). Publisher Correction: Microglial activation and tau propagate jointly across Braak stages. Nat. Med..

[53] Peng G., Qiu J., Liu H., Zhou M., Huang S., Guo W., Lin Y., Chen X., Li Z., Li G. (2020). Analysis of Cerebrospinal Fluid Soluble TREM2 and Polymorphisms in Sporadic Parkinson’s Disease in a Chinese Population. J. Mol. Neurosci..

[54] Roos P., von Essen M.R., Nielsen T.T., Johannsen P., Stokholm J., Bie A.S., Waldemar G., Simonsen A.H., Heslegrave A., Zetterberg H. (2018). Inflammatory markers of CHMP2B-mediated frontotemporal dementia. J. Neuroimmunol..

[55] Diaz-Lucena D., Kruse N., Thüne K., Schmitz M., Villar-Piqué A., da Cunha J.E.G., Hermann P., López-Pérez Ó., Andrés-Benito P., Ladogana A. (2021). TREM2 expression in the brain and biological fluids in prion diseases. Acta Neuropathol..

[56] Fabriek B.O., Moller H.J., Vloet R.P., van Winsen L.M., Hanemaaijer R., Teunissen C.E., Uitdehaag B.M., van den Berg T.K., Dijkstra C.D. (2007). Proteolytic shedding of the macrophage scavenger receptor CD163 in multiple sclerosis. J. Neuroimmunol..

[57] Housley W.J., Pitt D., Hafler D.A. (2015). Biomarkers in multiple sclerosis. Clin. Immunol..

[58] Ferraro D., Galli V., Vitetta F., Simone A.M., Bedin R., Del Giovane C., Morselli F., Filippini M.M., Nichelli P.F., Sola P. (2015). Cerebrospinal fluid CXCL13 in clinically isolated syndrome patients: Association with oligoclonal IgM bands and prediction of multiple sclerosis diagnosis. J. Neuroimmunol..

[59] De Fino C., Lucchini M., Lucchetti D., Nociti V., Losavio F.A., Bianco A., Colella F., Ricciardi-Tenore C., Sgambato A., Mirabella M. (2019). The predictive value of CSF multiple assay in multiple sclerosis: A single center experience. Mult. Scler. Relat. Disord..

[60] Zhong X., Wang H., Dai Y., Wu A., Bao J., Xu W., Cheng C., Lu Z., Qiu W., Hu X. (2011). Cerebrospinal fluid levels of CXCL13 are elevated in neuromyelitis optica. J. Neuroimmunol..

[61] Kimura A., Kishimoto T. (2010). IL-6: Regulator of Treg/Th17 balance. Eur. J. Immunol..

[62] Uzawa A., Mori M., Sato Y., Masuda S., Kuwabara S. (2012). CSF interleukin-6 level predicts recovery from neuromyelitis optica relapse. J. Neurol. Neurosurg. Psychiatry.

[63] Uzawa A., Mori M., Arai K., Sato Y., Hayakawa S., Masuda S., Taniguchi J., Kuwabara S. (2010). Cytokine and chemokine profiles in neuromyelitis optica: Significance of interleukin-6. Mult. Scler..

[64] Matsushita T., Tateishi T., Isobe N., Yonekawa T., Yamasaki R., Matsuse D., Murai H., Kira J. (2013). Characteristic cerebrospinal fluid cytokine/chemokine profiles in neuromyelitis optica, relapsing remitting or primary progressive multiple sclerosis. PLoS ONE.

[65] Kimura A., Takemura M., Saito K., Serrero G., Yoshikura N., Hayashi Y., Inuzuka T. (2017). Increased cerebrospinal fluid progranulin correlates with interleukin-6 in the acute phase of neuromyelitis optica spectrum disorder. J. Neuroimmunol..

[66] Wang Y., Zhou Y., Sun X., Lu T., Wei L., Fang L., Chen C., Huang Q., Hu X., Lu Z. (2016). Cytokine and Chemokine Profiles in Patients with Neuromyelitis Optica Spectrum Disorder. Neuroimmunomodulation.

[67] Zelek W.M., Fathalla D., Morgan A., Touchard S., Loveless S., Tallantyre E., Robertson N.P., Morgan B.P. (2020). Cerebrospinal fluid complement system biomarkers in demyelinating disease. Mult. Scler..

[68] Wang H.H., Dai Y.Q., Qiu W., Lu Z.Q., Peng F.H., Wang Y.G., Bao J., Li Y., Hu X.Q. (2011). Interleukin-17-secreting T cells in neuromyelitis optica and multiple sclerosis during relapse. J. Clin. Neurosci..

[69] Prins M., Dutta R., Baselmans B., Brevé J.J., Bol J.G., Deckard S.A., van der Valk P., Amor S., Trapp B.D., de Vries H.E. (2014). Discrepancy in CCL2 and CCR2 expression in white versus grey matter hippocampal lesions of Multiple Sclerosis patients. Acta Neuropathol. Commun..

[70] Moreira M.A., Souza A.L., Lana-Peixoto M.A., Teixeira M.M., Teixeira A.L. (2006). Chemokines in the cerebrospinal fluid of patients with active and stable relapsing-remitting multiple sclerosis. Braz. J. Med. Biol. Res..

[71] Malmeström C., Andersson B.A., Haghighi S., Lycke J. (2006). IL-6 and CCL2 levels in CSF are associated with the clinical course of MS: Implications for their possible immunopathogenic roles. J. Neuroimmunol..

[72] Westin K., Buchhave P., Nielsen H., Minthon L., Janciauskiene S., Hansson O. (2012). CCL2 is associated with a faster rate of cognitive decline during early stages of Alzheimer’s disease. PLoS ONE.

[73] Lycke J., Zetterberg H. (2017). The role of blood and CSF biomarkers in the evaluation of new treatments against multiple sclerosis. Expert Rev. Clin. Immunol..

[74] Axelsson M., Malmeström C., Nilsson S., Haghighi S., Rosengren L., Lycke J. (2011). Glial fibrillary acidic protein: A potential biomarker for progression in multiple sclerosis. J. Neurol..

[75] Lucchinetti C.F., Brück W., Rodriguez M., Lassmann H. (1996). Distinct patterns of multiple sclerosis pathology indicates heterogeneity on pathogenesis. Brain Pathol..

[76] Ozawa K., Suchanek G., Breitschopf H., Brück W., Budka H., Jellinger K., Lassmann H. (1994). Patterns of oligodendroglia pathology in multiple sclerosis. Brain.

[77] Sun M., Liu N., Xie Q., Li X., Sun J., Wang H., Wang M. (2021). A Candidate biomarker of glial fibrillary acidic protein in csf and blood in differentiating multiple sclerosis and its subtypes: A systematic review and meta-analysis. Mult. Scler. Relat. Disord..

[78] Petzold A., Eikelenboom M.J., Gveric D., Keir G., Chapman M., Lazeron R.H., Cuzner M.L., Polman C.H., Uitdehaag B.M., Thompson E.J. (2002). Markers for different glial cell responses in multiple sclerosis: Clinical and pathological correlations. Brain.

[79] Wei Y., Chang H., Li X., Wang H., Du L., Zhou H., Xu W., Ma Y., Yin L., Zhang X. (2018). Cytokines and Tissue Damage Biomarkers in First-Onset Neuromyelitis Optica Spectrum Disorders: Significance of Interleukin-6. Neuroimmunomodulation.

[80] Wei Y., Chang H., Li X., Du L., Xu W., Cong H., Yao Y., Zhang X., Yin L. (2018). CSF-S100B Is a Potential Candidate Biomarker for Neuromyelitis Optica Spectrum Disorders. Biomed. Res. Int..

[81] Kaneko K., Sato D.K., Nakashima I., Ogawa R., Akaishi T., Takai Y., Nishiyama S., Takahashi T., Misu T., Kuroda H. (2018). CSF cytokine profile in MOG-IgG+ neurological disease is similar to AQP4-IgG+ NMOSD but distinct from MS: A cross-sectional study and potential therapeutic implications. J. Neurol. Neurosurg. Psychiatry.

[82] Kaneko K., Sato D.K., Nakashima I., Nishiyama S., Tanaka S., Marignier R., Hyun J.W., Oliveira L.M., Reindl M., Seifert-Held T. (2016). Myelin injury without astrocytopathy in neuroinflammatory disorders with MOG antibodies. J. Neurol. Neurosurg. Psychiatry.

[83] Fujii C., Tokuda T., Ishigami N., Mizuno T., Nakagawa M. (2011). Usefulness of serum S100B as a marker for the acute phase of aquaporin-4 autoimmune syndrome. Neurosci. Lett..

[84] Misu T., Takano R., Fujihara K., Takahashi T., Sato S., Itoyama Y. (2009). Marked increase in cerebrospinal fluid glial fibrillar acidic protein in neuromyelitis optica: An astrocytic damage marker. J. Neurol. Neurosurg. Psychiatry.

[85] Takano R., Misu T., Takahashi T., Sato S., Fujihara K., Itoyama Y. (2010). Astrocytic damage is far more severe than demyelination in NMO: A clinical CSF biomarker study. Neurology.

[86] Petzold A., Marignier R., Verbeek M.M., Confavreux C. (2011). Glial but not axonal protein biomarkers as a new supportive diagnostic criteria for Devic neuromyelitis optica? Preliminary results on 188 patients with different neurological diseases. J. Neurol. Neurosurg. Psychiatry.

[87] Petzold A. (2005). Neurofilament phosphoforms: Surrogate markers for axonal injury, degeneration and loss. J. Neurol. Sci..

[88] Mattsson N., Cullen N.C., Andreasson U., Zetterberg H., Blennow K. (2019). Association between longitudinal plasma neurofilament light and neurodegeneration in patients with Alzheimer disease. JAMA Neurol..

[89] Khalil M., Teunissen C.E., Otto M., Piehl F., Sormani M.P., Gattringer T., Barro C., Kappos L., Comabella M., Fazekas F. (2018). Neurofilaments as biomarkers in neurological disorders. Nat. Rev. Neurol..

[90] Novakova L., Zetterberg H., Sundström P., Axelsson M., Khademi M., Gunnarsson M., Malmeström C., Svenningsson A., Olsson T., Piehl F. (2017). Monitoring disease activity in multiple sclerosis using serum neurofilament light protein. Neurology.

[91] Kuhle J., Kropshofer H., Hearing D.A., Kundu U., Meinert R., Barro C., Dahlke F., Tomic D., Leppert D., Kappos L. (2019). Blood neurofilament light chain as a biomarker of MS disease activity and treatment response. Neurology.

[92] Mariotto S., Gastaldi M., Grazian L., Mancinelli C., Capra R., Marignier R., Alberti D., Zanzoni S., Schanda K., Franciotta D. (2021). NfL levels predominantly increase at disease onset in MOG-Abs-associated disorders. Mult. Scler. Relat. Disord..

[93] Vakrakou A.G., Tzartos J.S., Strataki E., Boufidou F., Dimou E., Pyrgelis E.S., Constantinides V.C., Paraskevas G.P., Kapaki E. (2022). Neuronal and neuroaxonal damage cerebrospinal fluid biomarkers in autoimmune encephalitis associated or not with the presence of tumor. Biomedicines.

[94] Miyazawa I., Nakashima I., Petzold A., Fujihara K., Sato S., Itoyama Y. (2007). High CSF neurofilament heavy chain levels in neuromyelitis optica. Neurology.

[95] Wang H., Wang C., Qiu W., Lu Z., Hu X., Wang K. (2013). Cerebrospinal fluid light and heavy neurofilaments in neuromyelitis optica. Neurochem. Int..

[96] (2019). Lijun Peng, Chongfeng Bi, Deyu Xia, Linling Mao, Hairong Qian Increased cerebrospinal fluid neurofilament light chain in central nervous system inflammatory demyelinating disease. Mult. Scler. Relat. Disord..

[97] Pereira J.B., Westman E., Hansson O., Alzheimer’s Disease Neuroimaging Initiative (2017). Association between cerebrospinal fluid and plasma neurodegeneration biomarkers with brain atrophy in Alzheimer’s disease. Neurobiol. Aging.

[98] Bos I., Vos S., Verhey F., Scheltens P., Teunissen C., Engelborghs S., Sleegers K., Frisoni G., Blin O., Richardson J.C. (2019). Cerebrospinal fluid biomarkers of neurodegeneration, synaptic integrity, and astroglial activation across the clinical Alzheimer’s disease spectrum. Alzheimer’s Dement..

[99] Lehnert S., Costa J., de Carvalho M., Kirby J., Kuzma-Kozakiewicz M., Morelli C., Robberecht W., Shaw P., Silani V., Steinacker P. (2014). Multicentre quality control evaluation of different biomarker candidates for amyotrophic lateral sclerosis. Amyotroph. Lateral Scler. Frontotemporal. Degener..

[100] Oeckl P., Jardel C., Salachas F., Lamari F., Andersen P.M., Bowser R., de Carvalho M., Costa J., van Damme P., Gray E. (2016). Multicenter validation of CSF neurofilaments as diagnostic biomarkers for ALS. Amyotroph. Lateral Scler. Frontotemporal. Degener..

[101] Steinacker P., Feneberg E., Weishaupt J., Brettschneider J., Tumani H., Andersen P.M., von Arnim C.A., Böhm S., Kassubek J., Kubisch C. (2016). Neurofilaments in the diagnosis of motoneuron diseases: A prospective study on 455 patients. J. Neurol. Neurosurg. Psychiatry.

[102] Weydt P., Oeckl P., Huss A., Müller K., Volk A.E., Kuhle J., Knehr A., Andersen P.M., Prudlo J., Steinacker P. (2016). Neurofilament levels as biomarkers in asymptomatic and symptomatic familial amyotrophic lateral sclerosis. Ann. Neurol..

[103] Abu-Rumeileh S., Parchi P. (2021). Cerebrospinal fluid and blood neurofilament light chain protein in prion disease and other rapidly progressive dementias: Current state of the art. Front. Neurosci..

[104] Bernstein H.G., Baumann B., Danos P., Diekmann S., Bogerts B., Gundelfinger E.D., Braunewell K.H. (1999). Regional and cellular distribution of neural visinin-like protein immunoreactivities (VILIP-1 and VILIP-3) in human brain. J. Neurocytol..

[105] Schnurra I., Bernstein H.G., Riederer P., Braunewell K.H. (2001). The neuronal calcium sensor protein VILIP-1 is associated with amyloid plaques and extracellular tangles in Alzheimer’s disease and promotes cell death and tau phosphorylation in vitro: A link between calcium sensors and Alzheimer’s disease?. Neurobiol. Dis..

[106] Braunewell K.H. (2012). The visinin-like proteins VILIP-1 and VILIP-3 in Alzheimer’s disease-old wine in new bottles. Front. Mol. Neurosci..

[107] Zhang H., Ng K.P., Therriault J., Kang M.S., Pascoal T.A., Rosa-Neto P., Gauthier S., Alzheimer’s Disease Neuroimaging Initiative (2018). Cerebrospinal fluid phosphorylated tau, visinin-like protein-1, and chitinase-3-like protein 1 in mild cognitive impairment and Alzheimer’s disease. Transl. Neurodegener..

[108] Mavroudis I.A., Petridis F., Chatzikonstantinou S., Karantali E., Kazis D. (2021). A meta-analysis on the levels of VILIP-1 in the CSF of Alzheimer’s disease compared to normal controls and other neurodegenerative conditions. Aging Clin. Exp. Res..

[109] Tarawneh R., Lee J.M., Ladenson J.H., Morris J.C., Holtzman D.M. (2012). CSF VILIP-1 predicts rates of cognitive decline in early Alzheimer disease. Neurology.

[110] Tarawneh R., Head D., Allison S., Buckles V., Fagan A.M., Ladenson J.H., Morris J.C., Holtzman D.M. (2015). Cerebrospinal fluid markers of neurodegeneration and rates of brain atrophy in early Alzheimer disease. JAMA Neurol..

[111] Bishop P., Rocca D., Henley J.M. (2016). Ubiquitin C-terminal hydrolase L1 (UCH-L1): Structure, distribution and roles in brain function and dysfunction. Biochem. J..

[112] Setsuie R., Wada K. (2007). The functions of UCH-L1 and its relation to neurodegenerative diseases. Neurochem. Int..

[113] Song S., Jung Y.K. (2004). Alzheimer’s disease meets the ubiquitin-proteasome system. Trends Mol. Med..

[114] Xie M., Han Y., Yu Q., Wang X., Wang S., Liao X. (2016). UCH-L1 inhibition decreases the microtubule-binding function of tau protein. J. Alzheimer’s Dis..

[115] Öhrfelt A., Johansson P., Wallin A., Andreasson U., Zetterberg H., Blennow K., Svensson J. (2016). Increased Cerebrospinal Fluid Levels of Ubiquitin Carboxyl-Terminal Hydrolase L1 in Patients with Alzheimer’s Disease. Dement. Geriatr. Cogn. Dis. Extra.

[116] Barschke P., Oeckl P., Steinacker P., Al Shweiki M.R., Weishaupt J.H., Landwehrmeyer G.B., Anderl-Straub S., Weydt P., Diehl-Schmid J., Danek A. (2020). Different CSF protein profiles in amyotrophic lateral sclerosis and frontotemporal dementia with C9orf72 hexanucleotide repeat expansion. J. Neurol. Neurosurg. Psychiatry.

[117] Dobson R., Topping J., Davis A., Thompson E., Giovannoni G. (2013). Cerebrospinal fluid and urinary biomarkers in multiple sclerosis. Acta Neurol. Scand..

[118] Kester M.I., Teunissen C.E., Crimmins D.L., Herries E.M., Ladenson J.H., Scheltens P., van der Flier W.M., Morris J.C., Holtzman D.M., Fagan A.M. (2015). Neurogranin as a cerebrospinal fluid biomarker for synaptic loss in symptomatic Alzheimer disease. JAMA Neurol..

[119] Kvartsberg H., Duits F.H., Ingelsson M., Andreasen N., Öhrfelt A., Andersson K., Brinkmalm G., Lannfelt L., Minthon L., Hansson O. (2015). Cerebrospinal fluid levels of the synaptic protein neurogranin correlates with cognitive decline in prodromal Alzheimer’s disease. Alzheimer’s Dement..

[120] Wellington H., Paterson R.W., Portelius E., Törnqvist U., Magdalinou N., Fox N.C., Blennow K., Schott J.M., Zetterberg H. (2016). Increased CSF neurogranin concentration is specific to Alzheimer disease. Neurology.

[121] Portelius E., Olsson B., Höglund K., Cullen N.C., Kvartsberg H., Andreasson U., Zetterberg H., Sandelius Å., Shaw L.M., Lee V.M.Y. (2018). cerebrospinal fluid neurogranin concentration in neurodegeneration: Relation to clinical phenotypes and neuropathology. Acta Neuropathol..

[122] Brinkmalm A., Brinkmalm G., Honer W.G., Frölich L., Hausner L., Minthon L., Hansson O., Wallin A., Zetterberg H., Blennow K. (2014). SNAP-25 is a promising novel cerebrospinal fluid biomarker for synapse degeneration in Alzheimer’s disease. Mol. Neurodegener..

[123] Antonucci F., Corradini I., Fossati G., Tomasoni R., Menna E., Matteoli M. (2016). SNAP-25, a known presynaptic protein with emerging postsynaptic functions. Front. Synaptic Neurosci..

[124] Halbgebauer S., Steinacker P., Hengge S., Oeckl P., Rumeileh S.A., Anderl-Straub S., Lombardi J., Von Arnim C.A.F., Giese A., Ludolph A.C. (2022). CSF levels of SNAP-25 are increased early in Creutzfeldt-Jakob and Alzheimer’s disease. J. Neurol. Neurosurg. Psychiatry.

[125] Nilsson J., Ashton N.J., Benedet A.L., Montoliu-Gaya L., Gobom J., Pascoal T.A., Chamoun M., Portelius E., Jeromin A., Mendes M. (2022). Quantification of SNAP-25 with Mass Spectrometry and Simoa: A Method Comparison in Alzheimer’s Disease. Alzheimer’s Res. Ther..

[126] Milà-Alomà M., Brinkmalm A., Ashton N.J., Kvartsberg H., Shekari M., Operto G., Salvadó G., Falcon C., Gispert J.D., Vilor-Tejedor N. (2021). CSF Synaptic Biomarkers in the Preclinical Stage of Alzheimer Disease and Their Association with MRI and PET: A Cross-sectional Study. Neurology.

[127] Sutphen C.L., McCue L., Herries E.M., Xiong C., Ladenson J.H., Holtzman D.M., Fagan A.M., ADNI (2018). Longitudinal decreases in multiple cerebrospinal fluid biomarkers of neuronal injury in symptomatic late onset Alzheimer’s disease. Alzheimer’s Dement..

[128] Zhang H., Therriault J., Kang M.S., Ng K.P., Pascoal T.A., Rosa-Neto P., Gauthier S., Alzheimer’s Disease Neuroimaging Initiative (2018). Cerebrospinal fluid synaptosomal-associated protein 25 is a key player in synaptic degeneration in mild cognitive impairment and Alzheimer’s disease. Alzheimer’s Res. Ther..

[129] Neve R.L., Finch E.A., Bird E.D., Benowitz L.I. (1988). Growth-associated protein GAP-43 is expressed selectively in associative regions of the adult human brain. Proc. Natl. Acad. Sci. USA.

[130] Andersson A., Remnestål J., Nellgård B., Vunk H., Kotol D., Edfors F., Uhlén M., Schwenk J.M., Ilag L.L., Zetterberg H. (2019). Development of parallel reaction monitoring assays for cerebrospinal fluid proteins associated with Alzheimer’s disease. Clin. Chim. Acta.

[131] Remnestål J., Just D., Mitsios N., Fredolini C., Mulder J., Schwenk J.M., Uhlén M., Kultima K., Ingelsson M., Kilander L. (2016). CSF profiling of the human brain enriched proteome reveals associations of neuromodulin and neurogranin to Alzheimer’s disease. Proteom. Clin. Appl..

[132] Lan G., Cai Y., Li A., Liu Z., Ma S., Guo T., Alzheimer’s Disease Neuroimaging Initiative (2022). Association of presynaptic loss with Alzheimer’s disease and cognitive decline. Ann. Neurol..

[133] Sandelius Å., Sandgren S., Axelsson M., Malmeström C., Novakova L., Kostanjevecki V., Vandijck M., Blennow K., Zetterberg H., Lycke J. (2019). Cerebrospinal fluid growth-associated protein 43 in multiple sclerosis. Sci. Rep..

[134] Rot U., Sandelius Å., Emeršič A., Zetterberg H., Blennow K. (2018). Cerebrospinal fluid GAP-43 in early multiple sclerosis. Mult. Scler. J. Exp. Transl. Clin..

[135] Constantinides V.C., Paraskevas G.P., Emmanouilidou E., Petropoulou O., Bougea A., Vekrellis K., Evdokimidis I., Stamboulis E., Kapaki E. (2017). CSF biomarkers β-amyloid, Tau proteins and a-synuclein in the differential diagnosis of Parkinson-plus syndromes. J. Neurol. Sci..

[136] Kapaki E., Paraskevas G.P., Emmanouilidou E., Vekrellis K. (2013). The diagnostic value of CSF α-synuclein in the differential diagnosis of dementia with Lewy bodies vs. normal subjects and patients with Alzheimer’s disease. PLoS ONE.

[137] Rossi M., Candelise N., Baiardi S., Capellari S., Giannini G., Orrù C.D., Antelmi E., Mammana A., Hughson A.G., Calandra-Buonaura G. (2020). Ultrasensitive RT-QuIC assay with high sensitivity and specificity for Lewy body-associated synucleinopathies. Acta Neuropathol..

[138] Quadalti C., Calandra-Buonaura G., Baiardi S., Mastrangelo A., Rossi M., Zenesini C., Giannini G., Candelise N., Sambati L., Polischi B. (2021). Neurofilament light chain and α-synuclein RT-QuIC as differential diagnostic biomarkers in parkinsonisms and related syndromes. NPJ Park. Dis..

[139] Poggiolini I., Gupta V., Lawton M., Lee S., El-Turabi A., Querejeta-Coma A., Trenkwalder C., Sixel-Döring F., Foubert-Samier A., Pavy-Le Traon A. (2022). Diagnostic value of cerebrospinal fluid alpha-synuclein seed quantification in synucleinopathies. Brain.

[140] Goldman J.S., Farmer J.M., Wood E.M., Johnson J.K., Boxer A., Neuhaus J., Lomen-Hoerth C., Wilhelmsen K.C., Lee V.M., Grossman M. (2005). Comparison of family histories in FTLD subtypes and related tauopathies. Neurology.

[141] Neumann M., Sampathu D.M., Kwong L.K., Truax A.C., Micsenyi M.C., Chou T.T., Bruce J., Schuck T., Grossman M., Clark C.M. (2006). Ubiquitinated TDP-43 in frontotemporal lobar degeneration and amyotrophic lateral sclerosis. Science.

[142] Mackenzie I.R., Rademakers R. (2007). The molecular genetics and neuropathology of frontotemporal lobar degeneration: Recent developments. Neurogenetics.

[143] Sreedharan J., Blair I.P., Tripathi V.B., Hu X., Vance C., Rogelj B., Ackerley S., Durnall J.C., Williams K.L., Buratti E. (2008). TDP-43 mutations in familial and sporadic amyotrophic lateral sclerosis. Science.

[144] Xu F., Huang S., Li X.Y., Lin J., Feng X., Xie S., Wang Z., Li X., Zhu J., Lai H. (2022). Identification of TARDBP Gly298Ser as a founder mutation for amyotrophic lateral sclerosis in Southern China. BMC Med. Genom..

[145] Hasegawa M., Arai T., Nonaka T., Kametani F., Yoshida M., Hashizume Y., Beach T.G., Buratti E., Baralle F., Morita M. (2008). Phosphorylated TDP-43 in frontotemporal lobar degeneration and amyotrophic lateral sclerosis. Ann. Neurol..

[146] Steinacker P., Hendrich C., Sperfeld A.D., Jesse S., von Arnim C.A., Lehnert S., Pabst A., Uttner I., Tumani H., Lee V.M. (2008). TDP-43 in cerebrospinal fluid of patients with frontotemporal lobar degeneration and amyotrophic lateral sclerosis. Arch. Neurol..

[147] Kasai T., Tokuda T., Ishigami N., Sasayama H., Foulds P., Mitchell D.J., Mann D.M., Allsop D., Nakagawa M. (2009). Increased TDP-43 protein in cerebrospinal fluid of patients with amyotrophic lateral sclerosis. Acta Neuropathol..

[148] Junttila A., Kuvaja M., Hartikainen P., Siloaho M., Helisalmi S., Moilanen V., Kiviharju A., Jansson L., Tienari P.J., Remes A.M. (2016). Cerebrospinal fluid TDP-43 in frontotemporal lobar degeneration and amyotrophic lateral sclerosis patients with and without the C9ORF72 hexanucleotide expansion. Dement. Geriatr. Cogn. Dis. Extra.

[149] Kapaki E., Boufidou F., Bourbouli M., Pyrgelis E.S., Constantinides V.C., Anastassopoulou C., Paraskevas G.P. (2022). Cerebrospinal fluid biomarker profile in TDP-43-related genetic frontotemporal dementia. J. Pers. Med..

[150] Bourbouli M., Paraskevas G.P., Rentzos M., Mathioudakis L., Zouvelou V., Bougea A., Tychalas A., Kimiskidis V.K., Constantinides V., Zafeiris S. (2021). Genotyping and plasma/cerebrospinal fluid profiling of a cohort of frontotemporal dementia-amyotrophic lateral sclerosis patients. Brain Sci..

[151] Bourbouli M., Rentzos M., Bougea A., Zouvelou V., Constantinides V.C., Zaganas I., Evdokimidis I., Kapaki E., Paraskevas G.P. (2017). Cerebrospinal fluid TAR DNA-binding protein 43 combined with tau proteins as a candidate biomarker for amyotrophic lateral sclerosis and frontotemporal dementia spectrum disorders. Dement. Geriatr. Cogn. Disord..

[152] Foulds P.G., Davidson Y., Mishra M., Hobson D.J., Humphreys K.M., Taylor M., Johnson N., Weintraub S., Akiyama H., Arai T. (2009). Plasma phosphorylated-TDP-43 protein levels correlate with brain pathology in frontotemporal lobar degeneration. Acta Neuropathol..

[153] Songsrirote K., Li Z., Ashford D., Bateman A., Thomas-Oates J. (2010). Development and application of mass spectrometric methods for the analysis of progranulin N-glycosylation. J. Proteom..

[154] Chitramuthu B.P., Bennett Hugh P.J., Bateman A. (2017). Progranulin: A new avenue towards the understanding and treatment of neurodegenerative disease. Brain.

[155] Zhou X., Kukar T., Rademakers R. (2021). Lysosomal dysfunction and other pathomechanisms in FTLD: Evidence from progranulin genetics and biology. Adv. Exp. Med. Biol..

[156] Van Damme P., van Hoecke A., Lambrechts D., Vanacker P., Bogaert E., van Swieten J., Carmeliet P., Van Den Bosch L., Robberecht W. (2008). Progranulin functions as a neurotrophic factor to regulate neurite outgrowth and enhance neuronal survival. J. Cell Biol..

[157] Petkau T.L., Leavitt B.R. (2014). Progranulin in neurodegenerative disease. Trends Neurosci..

[158] Tanaka Y., Chambers J.K., Matsuwaki T., Yamanouchi K., Nishihara M. (2014). Possible involvement of lysosomal dysfunction in pathological changes of the brain in aged progranulin-deficient mice. Acta Neuropathol. Commun..

[159] Lui H., Zhang J., Makinson S.R., Cahill M.K., Kelley K.W., Huang H.Y., Shang Y., Oldham M.C., Martens L.H., Gao F. (2016). Progranulin deficiency promotes circuit-specific synaptic pruning by microglia via complement activation. Cell.

[160] Baker M., Mackenzie I.R., Pickering-Brown S.M., Gass J., Rademakers R., Lindholm C., Snowden J., Adamson J., Sadovnick A.D., Rollinson S. (2006). Mutations in progranulin cause tau-negative frontotemporal dementia linked to chromosome 17. Nature.

[161] Rademakers R., Neumann M., Mackenzie I.R. (2012). Advances in understanding the molecular basis of frontotemporal dementia. Nat. Rev. Neurol..

[162] Perry D.C., Lehmann M., Yokoyama J.S., Karydas A., Lee J.J., Coppola G., Grinberg L.T., Geschwind D., Seeley W.W., Miller B.L. (2013). Progranulin mutations as risk factors for Alzheimer disease. JAMA Neurol..

[163] Sieben A., van Langenhove T., Engelborghs S., Martin J.J., Boon P., Cras P., de Deyn P.P., Santens P., van Broeckhoven C., Cruts M. (2012). The genetics and neuropathology of frontotemporal lobar degeneration. Acta Neuropathol..

[164] Meeter L.H., Patzke H., Loewen G., Dopper E.G., Pijnenburg Y.A., van Minkelen R., van Swieten J.C. (2016). Progranulin Levels in Plasma and Cerebrospinal Fluid in Granulin Mutation Carriers. Dement. Geriatr. Cogn. Dis. Extra.

[165] Galimberti D., Bonsi R., Fenoglio C., Serpente M., Cioffi S.M., Fumagalli G., Arighi A., Ghezzi L., Arcaro M., Mercurio M. (2015). Inflammatory molecules in Frontotemporal Dementia: Cerebrospinal fluid signature of progranulin mutation carriers. Brain Behav. Immun..

[166] Wilke C., Gillardon F., Deuschle C., Hobert M.A., Jansen I.E., Metzger F.G., Heutink P., Gasser T., Maetzler W., Blauwendraat C. (2017). Cerebrospinal Fluid Progranulin, but Not Serum Progranulin, Is Reduced in GRN-Negative Frontotemporal Dementia. Neurodegener. Dis..

[167] Morenas-Rodríguez E., Cervera-Carles L., Vilaplana E., Alcolea D., Carmona-Iragui M., Dols-Icardo O., Ribosa-Nogué R., Muñoz-Llahuna L., Sala I., Belén Sánchez-Saudinós M. (2016). Progranulin Protein Levels in Cerebrospinal Fluid in Primary Neurodegenerative Dementias. J. Alzheimer’s Dis..

[168] Pawlitzki M., Sweeney-Reed C.M., Bittner D., Lux A., Vielhaber S., Schreiber S., Paul F., Neumann J. (2018). CSF-progranulin and neurofilament light chain levels in patients with radiologically isolated syndrome-sign of inflammation. Front. Neurol..

[169] De Riz M., Galimberti D., Fenoglio C., Piccio L.M., Scalabrini D., Venturelli E., Pietroboni A., Piola M., Naismith R.T., Parks B.J. (2010). Cerebrospinal fluid progranulin levels in patients with different multiple sclerosis subtypes. Neurosci. Lett..

[170] Vercellino M., Grifoni S., Romagnolo A., Masera S., Mattioda A., Trebini C., Chiavazza C., Caligiana L., Capello E., Mancardi G.L. (2011). Progranulin expression in brain tissue and cerebrospinal fluid levels in multiple sclerosis. Mult. Scler..

[171] Khan I., Preeti K., Fernandes V., Khatri D.K., Singh S.B. (2022). Role of MicroRNAs, Aptamers in Neuroinflammation and Neurodegenerative Disorders. Cell Mol. Neurobiol..

[172] Selmaj K.W., Mycko M.P., Furlan R., Rejdak K. (2022). Fluid phase biomarkers in multiple sclerosis. Curr. Opin. Neurol..

[173] Mandolesi G., Rizzo F.R., Balletta S., Stampanoni Bassi M., Gilio L., Guadalupi L., Nencini M., Moscatelli A., Ryan C.P., Licursi V. (2021). The microRNA let-7b-5p Is Negatively Associated with Inflammation and Disease Severity in Multiple Sclerosis. Cells.

[174] De Vito F., Musella A., Fresegna D., Rizzo F.R., Gentile A., Stampanoni Bassi M., Gilio L., Buttari F., Procaccini C., Colamatteo A. (2022). MiR-142-3p regulates synaptopathy-driven disease progression in multiple sclerosis. Neuropathol. Appl. Neurobiol..

[175] Su Y., Li Z., Rang X., Wang Y., Fu J. (2022). Integrated Analysis and Identification of CSF-Derived Risk miRNAs and Pivotal Genes in Multiple Sclerosis. J. Mol. Neurosci..

[176] Ahlbrecht J., Martino F., Pul R., Skripuletz T., Sühs K.W., Schauerte C., Yildiz Ö., Trebst C., Tasto L., Thum S. (2016). Deregulation of microRNA-181c in cerebrospinal fluid of patients with clinically isolated syndrome is associated with early conversion to relapsing-remitting multiple sclerosis. Mult. Scler..

[177] Bergman P., Piket E., Khademi M., James T., Brundin L., Olsson T., Piehl F., Jagodic M. (2016). Circulating miR-150 in CSF is a novel candidate biomarker for multiple sclerosis. Neurol. Neuroimmunol. Neuroinflamm..

[178] Perdaens O., Dang H.A., D’Auria L., van Pesch V. (2020). CSF microRNAs discriminate MS activity and share similarity to other neuroinflammatory disorders. Neurol. Neuroimmunol. Neuroinflamm..

[179] Zheleznyakova G.Y., Piket E., Needhamsen M., Hagemann-Jensen M., Ekman D., Han Y., James T., Khademi M., Al Nimer F., Scicluna P. (2021). Small noncoding RNA profiling across cellular and biofluid compartments and their implications for multiple sclerosis immunopathology. Proc. Natl. Acad. Sci. USA.

[180] Sala Frigerio C., Lau P., Salta E., Tournoy J., Bossers K., Vandenberghe R., Wallin A., Bjerke M., Zetterberg H., Blennow K. (2013). Reduced expression of hsa-miR-27a-3p in CSF of patients with Alzheimer disease. Neurology.

[181] Burgos K., Malenica I., Metpally R., Courtright A., Rakela B., Beach T., Shill H., Adler C., Sabbagh M., Villa S. (2014). Profiles of extracellular miRNA in cerebrospinal fluid and serum from patients with Alzheimer’s and Parkinson’s diseases correlate with disease status and features of pathology. PLoS ONE.

[182] Kiko T., Nakagawa K., Tsuduki T., Furukawa K., Arai H., Miyazawa T. (2014). MicroRNAs in plasma and cerebrospinal fluid as potential markers for Alzheimer’s disease. J. Alzheimer’s Dis..

[183] Müller M., Jäkel L., Bruinsma I.B., Claassen J.A., Kuiperij H.B., Verbeek M.M. (2016). MicroRNA-29a is a candidate biomarker for Alzheimer’s disease in cell-free cerebrospinal fluid. Mol. Neurobiol..

[184] Sørensen S.S., Nygaard A.B., Christensen T. (2016). miRNA expression profiles in cerebrospinal fluid and blood of patients with Alzheimer’s disease and other types of dementia—An exploratory study. Transl. Neurodegener..

[185] Starhof C., Hejl A.M., Heegaard N.H.H., Carlsen A.L., Burton M., Lilje B., Winge K. (2019). The biomarker potential of cell-free microrna from cerebrospinal fluid in parkinsonian syndromes. Mov. Disord..

[186] De Felice B., Annunziata A., Fiorentino G., Borra M., Biffali E., Coppola C., Cotrufo R., Brettschneider J., Giordana M.L., Dalmay T. (2014). miR-338-3p is over-expressed in blood, CFS, serum and spinal cord from sporadic amyotrophic lateral sclerosis patients. Neurogenetics.

[187] Chandrananda D., Thorne N.P., Bahlo M. (2015). High-resolution characterization of sequence signatures due to non-random cleavage of cell-free DNA. BMC Med. Genom..

[188] Pan W., Gu W., Nagpal S., Gephart M.H., Quake S.R. (2015). Brain tumor mutations detected in cerebral spinal fluid. Clin. Chem..

[189] Wang Y., Springer S., Zhang M., McMahon K.W., Kinde I., Dobbyn L., Ptak J., Brem H., Chaichana K., Gallia G.L. (2015). Detection of tumor-derived DNA in cerebrospinal fluid of patients with primary tumors of the brain and spinal cord. Proc. Natl. Acad. Sci. USA.

[190] García-Romero N., Carrión-Navarro J., Areal-Hidalgo P., Ortiz de Mendivil A., Asensi-Puig A., Madurga R., Núñez-Torres R., González-Neira A., Belda-Iniesta C., González-Rumayor V. (2019). BRAF V600E detection in liquid biopsies from pediatric central nervous system tumors. Cancers.

[191] Park J.S., Lee J., Jung E.S., Kim M.H., Kim I.B., Son H., Kim S., Kim S., Park Y.M., Mook-Jung I. (2019). Brain somatic mutations observed in Alzheimer’s disease associated with aging and dysregulation of tau phosphorylation. Nat. Commun..

[192] Pai M.C., Kuo Y.M., Wang I.F., Chiang P.M., Tsai K.J. (2019). The role of methylated circulating nucleic acids as a potential biomarker in Alzheimer’s disease. Mol. Neurobiol..

[193] Bahado-Singh R.O., Radhakrishna U., Gordevičius J., Aydas B., Yilmaz A., Jafar F., Imam K., Maddens M., Challapalli K., Metpally R.P. (2022). Artificial intelligence and circulating cell-free DNA methylation profiling: Mechanism and detection of Alzheimer’s disease. Cells.

[194] Lehmann-Werman R., Neiman D., Zemmour H., Moss J., Magenheim J., Vaknin-Dembinsky A., Rubertsson S., Nellgård B., Blennow K., Zetterberg H. (2016). Identification of tissue-specific cell death using methylation patterns of circulating dna. Proc. Natl. Acad. Sci. USA.

[195] Mendioroz M., Martínez-Merino L., Blanco-Luquin I., Urdánoz A., Roldán M., Jericó I. (2018). Liquid biopsy: A new source of candidate biomarkers in amyotrophic lateral sclerosis. Ann. Clin. Transl. Neurol..

[196] Caggiano C., Celona B., Garton F., Mefford J., Black B.L., Henderson R., Lomen-Hoerth C., Dahl A., Zaitlen N. (2021). Comprehensive cell type decomposition of circulating cell-free DNA with CelFiE. Nat. Commun..

[197] Grazioli S., Pugin J. (2018). Mitochondrial damage-associated molecular patterns: From inflammatory signaling to human diseases. Front. Immunol..

[198] Lowes H., Pyle A., Duddy M., Hudson G. (2019). Cell-free mitochondrial DNA in progressive multiple sclerosis. Mitochondrion.

[199] Leurs C.E., Podlesniy P., Trullas R., Balk L., Steenwijk M.D., Malekzadeh A., Piehl F., Uitdehaag B.M., Killestein J., van Horssen J. (2018). Cerebrospinal fluid mtDNA concentration is elevated in multiple sclerosis disease and responds to treatment. Mult. Scler..

[200] Podlesniy P., Figueiro-Silva J., Llado A., Antonell A., Sanchez-Valle R., Alcolea D., Lleo A., Molinuevo J.L., Serra N., Trullas R. (2013). Low cerebrospinal fluid concentration of mitochondrial DNA in preclinical Alzheimer disease. Ann. Neurol..

[201] Pyle A., Brennan R., Kurzawa-Akanbi M., Yarnall A., Thouin A., Mollenhauer B., Burn D., Chinnery P.F., Hudson G. (2015). Reduced cerebrospinal fluid mitochondrial DNA is a biomarker for early-stage Parkinson’s disease. Ann. Neurol..

[202] Lowes H., Pyle A., Santibanez-Koref M., Hudson G. (2020). Circulating cell-free mitochondrial DNA levels in Parkinson’s disease are influenced by treatment. Mol. Neurodegener..

